# Understanding stakeholders’ perceptions of the impact of extractive industries on adolescent health and well-being in Mozambique: a qualitative study

**DOI:** 10.1136/bmjopen-2024-088207

**Published:** 2025-06-06

**Authors:** Olga Cambaco, Christoff Galvão, Curdin Brugger, Khátia R Munguambe, Jürg Utzinger, Astrid M Knoblauch, Mirko S Winkler

**Affiliations:** 1 Swiss Tropical and Public Health Institute, Allschwil, Switzerland; 2 University of Basel, Basel, Switzerland; 3 Manhiça Health Research Centre, Maputo, Mozambique; 4 Faculty of Medicine, Eduardo Mondlane University, Maputo, Mozambique

**Keywords:** Adolescents, Health, PUBLIC HEALTH, QUALITATIVE RESEARCH

## Abstract

**Abstract:**

**Objectives:**

Adolescent health is critical for achieving Sustainable Development Goal 3, ‘health for all at all ages’. In sub-Saharan Africa (SSA), mining projects hold promise for social and economic development. Yet, the extent to which the health and well-being of adolescents are impacted by industrial mining is poorly understood. In this paper, we aim to investigate how adolescent health and well-being is perceived by key informants and caregivers in communities that have been affected by industrial mining projects in Mozambique.

**Design and settings:**

A qualitative study was implemented from May–July 2022 in two rural districts in Mozambique’s northern and central regions. Both districts have large industrial mining projects: a coal mine in Moatize district and a mineral sands mine in Moma district.

**Participants:**

A total of 21 key informant interviews (KIIs) were conducted with a range of stakeholders, including health professionals and civil society and private sector representatives. In addition, four focus group discussions (FGDs) were conducted with adolescents’ caregivers.

**Results:**

Through the combined results from the KIIs and FGDs, four main health concerns affecting adolescents in mining areas were identified: sexually transmitted infections, respiratory tract infections, diarrhoeal diseases and malaria. Mining activities were perceived to exacerbate negative health effects and contribute to poor health outcomes among adolescents. Although mining companies invested in public infrastructure, most participants did not perceive this investment as a positive contribution to the health and well-being of adolescents.

**Conclusion:**

Our study sheds light on the multifaceted challenges perceived by stakeholders that adolescents residing in mining communities in Mozambique face. Insufficient priority is given to effective interventions that specifically target adolescent health in the two study mining areas. In order to leave no one behind, as stipulated by the sustainable development agenda, more emphasis should be placed on the role and responsibility of mining companies in adequately addressing adolescents’ unique health needs in mining settings in SSA.

**STRENGTHS AND LIMITATIONS OF THIS STUDY**

- The strengths of the study rely on its capability to include in-depth insights and perspectives of those who care for, make decisions on behalf of and serve adolescents (caregivers and multiple key informants from government staff, the private sector, non-governmental organisations and civil society), providing contextually relevant findings to inform policy and practices.
- The findings reflect caregivers’ and different stakeholders’ perceptions and may not necessarily align with the actual experiences of adolescents themselves in mining areas.
- The study only focused on two specific mining sites at a single point in time. Hence, the findings may not be generalisable to all mining areas in Mozambique and beyond.
- The findings from the mining sector representatives are limited to the perspective of one participant, which limits the depth and richness of the analysis.
- By focusing on specific groups, the study may not fully capture the complexity of the issue, as it does not account for broader external factors, such as national policies or economic conditions, which could influence the experiences of caregivers and stakeholders.

## Introduction

 According to the World Health Organization (WHO), adolescents are individuals aged 10–19 years, representing a critical stage of physical, cognitive, emotional and social development.^1^ There are approximately 1.2 billion adolescents, thereby representing 18% of the global population.^2^ Nearly 90% of the adolescents live in countries with a low human development index (HDI).^3^ It is the largest cohort of this age group in human history; yet, the number of adolescents is expected to continue to grow until 2050.^4^ In 2023, road injuries, diarrhoeal diseases and tuberculosis ranked as the three leading causes for disability-adjusted life years in adolescents worldwide. Interpersonal violence, self-harm, congenital anomalies, HIV/AIDS, lower respiratory tract infections, epilepsy and drowning are other major issues affecting adolescents’ health.^5^ In addition, the transition into adulthood is characterised by challenges that might contribute to poor health in adolescents, including aspects such as self-esteem and autonomy, along with different forms of socialisation (eg, social capital, social support and local networks), which have the potential to jeopardise the physical, social and mental health and well-being of adolescents.^6^

In sub-Saharan Africa (SSA), the situation of adolescents is of particular concern due to the rapid growth of this population group, coupled with the high burden and specific disease profiles in this age group.^7^ Socially and economically disadvantaged adolescents encounter particular challenges due to poverty, illiteracy and unemployment, as well as power and structural inequalities.^8 9^ Mozambique’s colonial and postcolonial history has played a significant role in shaping the structural inequalities that impact both resource extraction-driven economic development and the availability of health services.^10^ Following independence from Portugal, Mozambique initially pursued a strong primary healthcare approach.^11^ However, foreign military interventions and macroeconomic reforms—particularly those tied to structural adjustment programmes—severely weakened the health system, leading to decades of austerity and reduced access to essential services.^12^ In addition to Pfeiffer’s work, scholars such as Hanlon and Pitcher have examined how Mozambique’s economic liberalisation and privatisation policies, driven by structural adjustment programmes, have exacerbated inequalities in access to health services.^13 14^ These reforms, while intended to stabilise the economy, often led to the underfunding of public services, disproportionately affecting vulnerable populations, including those in resource extraction zones.^11^ Understanding these historical and economic dynamics is critical for framing discussions on health equity and the role of the state versus corporate actors in ensuring access to healthcare for mining-affected communities, particularly to the most marginalised and vulnerable, including adolescents.^15^ In fact, in Mozambique, some mining companies have engaged in corporate social responsibility (CSR) initiatives aimed at addressing community health concerns, including access to healthcare and environmental impact mitigation. However, these efforts often primarily target adults and younger children, with adolescents being frequently overlooked in programme design and implementation. Rather than relying solely on voluntary CSR initiatives from mining companies, addressing these disparities requires a more sustainable approach.^16^ Additionally, religious and cultural norms increase vulnerability and/or marginalisation of adolescents.^17^,^21^ Consequently, adolescents’ health needs, behaviours and expectations are particular; yet, healthcare services are often inadequately equipped to address them.^22 23^ Hence, in many settings in SSA, adolescents are at disproportionately high risk of disease, poor well-being and trauma, posing a threat to a prosperous future.^18 22 24^

Mining projects are seen as an opportunity to promote social and economic growth in many countries of SSA.^25^ This will be further spurred by the shift to low carbon technologies, which is anticipated to result in a substantially increased demand for specific minerals and metals.^25 26^ Hence, the extractive industries represent an opportunity in SSA to further progress towards achieving the Sustainable Development Goals (SDGs), including SDG 3 (ie, ‘to ensure healthy lives and promote well-being for all at all ages’).^27^ On the other hand, a range of adverse health effects in communities affected by resource extraction projects has been documented in mining areas.^28^,^30^

While the health and well-being of adults and children living in industrial and small-scale mining areas have been studied relatively well,^31^,^34^ there is a paucity of studies that have investigated the health challenges faced by adolescents living in resource extraction contexts in SSA.^35 36^ Various studies found adolescent health outcomes in mining areas to be exacerbated.^16 30^ In Brazil, the authors highlight the significant perception of health risks faced by adolescents in gold mining regions due to mercury exposure.^37^ Studies in Canada identified four main ways in which the sociocultural and structural conditions created by the extractive industries are perceived by adolescents to affect them, including sexual behaviours, fuelling the spread of sexually transmitted infections (STIs), mobility of oil/gas workers, binge partying, high levels of disposable income and gendered power dynamics.^29 30^ Similarly, the qualitative research conducted by Leuneberger *et al*, in communities in SSA, including Mozambique, showed that communities perceived mainly negative impacts of large-scale mining on adolescents.^38^ Similar results were found in our previous study with adolescents conducted in two rural mining districts in Mozambique.^39^ In a context such as Mozambique, where adolescents (and particularly adolescent girls) face several hurdles to maintaining or regaining good health, mining exacerbates living conditions and perpetuates cycles of poverty and vulnerability.^40^ New research is needed to understand those issues better, especially understanding how the identified health outcomes translate into the daily lives of different strata of communities in mining areas and their strategies for managing their well-being, particularly among vulnerable subgroups such as adolescents in mining areas.^38 39^ Additionally, it is, for example, poorly documented whether decision-makers and health practitioners pay sufficient attention to adolescent health and well-being when developing and implementing health impact mitigation strategies in mining areas.^35^ Similarly, in Mozambique, little has been done to investigate policies and interventions for addressing public health and social issues that affect adolescents in mining communities. Qualitative studies are better in understanding and capturing both specific health needs and the perceptions about underlying determinants in resource extraction settings.^38^ To address the identified knowledge gap, we conducted a qualitative study to investigate health and well-being challenges perceived by key informants and caregivers in industrial rural areas in Mozambique. In addition, we investigated the extent to which mining companies engage in mitigating potential adolescent-specific health risks in impacted communities.

## Methods

### Study settings

**Figure 1:**
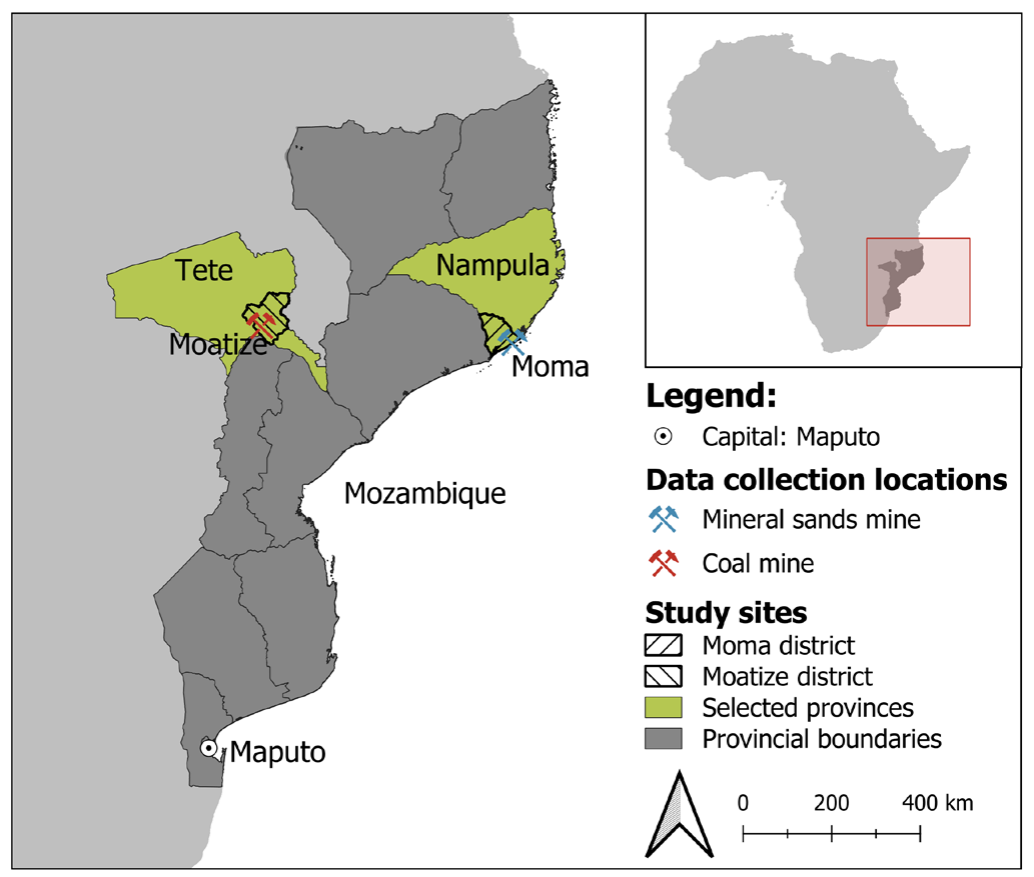
Study sites, Moma and Moatize districts, Mozambique, 2022.

The study was conducted in Moma and Moatize districts, located in Mozambique’s northern and central regions, respectively (figure 1). The districts were purposively selected based on the presence of large industrial mining projects, namely a coal mine in Moatize district and a mineral sands mine in Moma district.^41^ Both are mainly rural districts with low urbanisation rates. The primary occupations of the inhabitants include farming, forestry, fishery, livestock, trading and handicrafts.^42 43^Adolescents represent approximately 8 million people, accounting for 25% of of Mozambique's total population. Specifically, the population of adolescents in Moma is 81 110, and in Moatize, it is 85 886.^42 43^ The fertility rates in these districts are high, with 5.1 and 6.9 children per women, while child mortality rates are at 85 and 126 deaths per 1000 live births, respectively, and thus, above the provincial average.^44 45^ With only one health facility per 24 000 people in Moma and 29 000 people in Moatize, health service coverage is low.^44 45^

### Design

**Figure 2:**
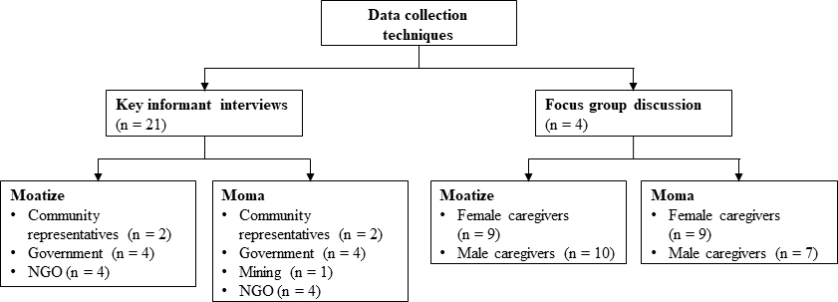
Key informant interviews and focus group discussions with key informants and caregivers, Moma and Moatize districts, Mozambique, 2022, NGO, non-governmental organisation.

A cross-sectional study design was employed, using qualitative data collection techniques, namely focus group discussions (FGDs) and key informant interviews (KIIs) (figure 2). This approach provides an indepth insight into key informants’ and caregivers’ perspectives and experiences, enabling a more comprehensive understanding of specific health issues in target populations.^46 47^ Data on adolescents’ experiences and perceived health impacts of living in rural mining areas in Mozambique were also collected and have been presented elsewhere.^39^

### Participants

The study population consisted of individuals who possessed first-hand knowledge, work experience and current involvement in adolescent health, as well as adult caregivers of adolescents living in the selected mining areas. Notably, the perspectives of young people, particularly those aged 18–19 years, who assumed caregiving responsibilities due to various social or economic circumstances, were not captured in this study but were included in the main study.^39^ In our research, stakeholders refer to government and private sector representatives, civil society organisations, mining company representatives, caregivers (of adolescents aged 10–19 years) and other local individuals who have a direct or indirect interest in or inflluence on the health and well-being of adolescents in communities affected by extractive industries. The specific nature of the stake they hold varies according to each group’s distinct interests, obligations and contribution, shaping their engagement in different ways.

To obtain an overview of the study sites, a transect walk was conducted under the guidance of the community leaders prior to data collection.^38^ This approach allowed the research team to identify eligible participants for the FGDs with high fidelity and understand the context in which adolescents lived. Community leaders were approached to help identify the caregivers of adolescents and encouraged to make efforts to maximise the diversity among eligible participants. The following inclusion criteria were defined for the caregivers of adolescents: (1) community members willing and able to express themselves in a group; (2) female and male caregivers of adolescents living in the selected mining areas; and (3) caregivers living in the geographical area where the FGDs were held but not from the same household.

With the aim of capturing different perspectives across government and non-government levels, including district and community levels, key informants were purposively selected based on the following inclusion criteria: (1) living and/or working in the local mining communities and (2) health professionals or individuals whose work or occupation is related to the health of adolescents. Key informants representing non-governmental organisations (NGOs), the health sector, the education sector, community leaders, civil society organisations working in adolescent health and mining companies were invited for the interviews.

### Data collection

The KIIs and FGDs were conducted between May and July 2022. A semistructured guide for KIIs and FGDs (see online supplemental files: Key informants and FGDs Caregivers) was designed based on the main objective of the study and piloted before use. The KIIs and FGDs were facilitated by social science assistants who are trained in qualitative research, with support from the principal investigator, who is a qualitative researcher (OC). KIIs were conducted face-to-face at the workplace or in the community (online supplemental figure). In case the participant’s schedule did not allow an in-person meeting, the interview was conducted by mobile phone.

FGDs with adolescents’ caregivers were conducted in community halls or schools, with 7–10 participants and two FGDs in each district (online supplemental figure). The participants agreed on the locations, and the research team ensured sufficient privacy and comfort. Prior to the KIIs and FGDs, all participants were informed about the objectives of the study and data collection procedures. Written informed consent was obtained from each participant. Participation was voluntary and an individual could withdraw any time without further obligation. The KIIs and FGDs were conducted in Portuguese or the local languages spoken (ie, Makua or Nhungue), depending on the participant’s preferences, and were translated and transcribed into Portuguese for analysis. All KIIs and FGDs were audiorecorded using digital voice recorders.

### Data management and analysis

Audiorecorded data from KIIs and FGDs were transcribed *verbatim* and uploaded onto a password-protected computer for anonymised transcription by the social science assistants. To assure transcription quality, a random sample of transcripts (20%) was checked for accuracy and approved by the principal investigator (OC). In order to ensure consistency and accuracy, the most experienced team member (encoder) carried out quality control of all the transcripts by comparing the audio with the transcription to identify and remove any errors that might have occurred.

To identify the most prevalent themes discussed by the participants, the transcripts were analysed manually using a qualitative thematic analysis approach.^48^ An initial coding scheme was created by the principal investigator (OC) based on the study objectives. A matrix was designed by OC and revised by CG, where the rows represented the participants’ ID and the columns the questions addressed (organised into predetermined and emerging themes), which oriented a process of abductive coding. Once all the data were coded, they were summarised in a matrix for each theme and inserted into a matrix using Microsoft Excel (OC and CG). This process allowed for the development of emergent themes from the data, consistent with an inductive coding process. Four main themes emerged from the analysis, which were analysed based on their contextual importance during the KIIs and FGDs.

**Figure 3:**
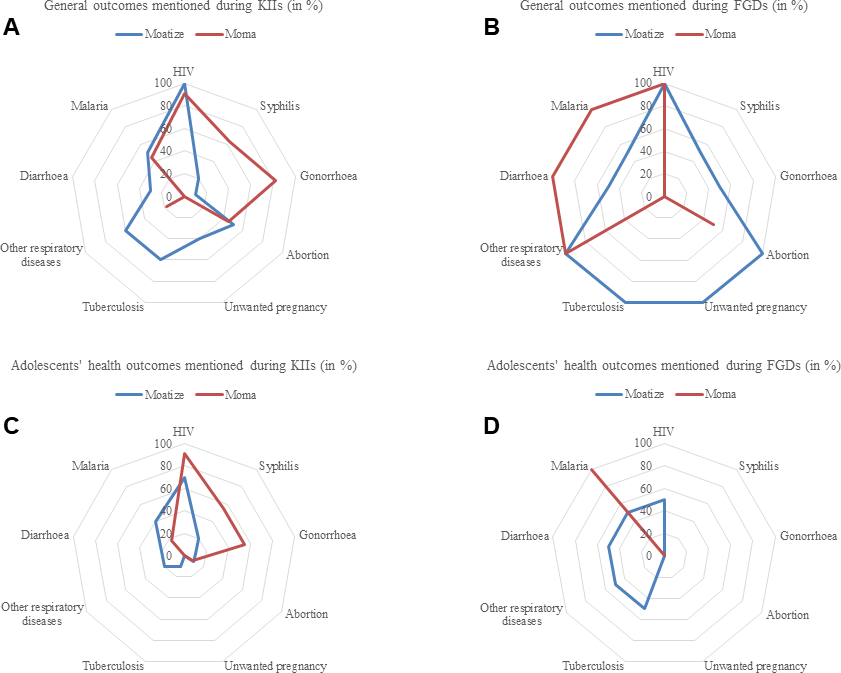
Adolescent health outcomes, Moma and Moatize districts, Mozambique, 2022. Graphs (A) and (B) illustrate general health outcomes mentioned in the key informant interviews (KIIs) and focus group discussions (FGDs) with caregivers. Graphs (C) and (D) illustrate the perceived adolescents’ health outcomes affecting adolescents.

Furthermore, based on the health outcomes framework, perceived health conditions were systematically categorised. The framework helped the investigator (OC) to consider different health outcomes on communities, including self-assessed health status categories.^49^ Therefore, each health condition mentioned by participants during interviews and group discussions was typed in a separate Microsoft Excel sheet, where each mentioned health outcome had a different category. For individual health outcomes, the number of times each category occurred was counted and presented in frequency for each KII and FGD. The percentages were calculated based on the frequency, ranging from 0% to 100%, with 100% indicating that all KIIs or FGDs mentioned the specific health outcome, while 0% indicated that none of the KIIs or FGDs mentioned the health outcome. By comparing the frequency of mentioned health outcomes between KIIs and FGDs and study sites, we aimed to identify any discrepancies or similarities in the perceived health outcomes. Therefore, proportions are graphically presented using a radar web chart (figure 3).

The comparison made between the different participants’ backgrounds, organisational affiliations and mining settings allowed for a comprehensive understanding of the diverse perspectives and experiences brought by different individuals involved in the study. However, one interview from a mining company representative was excluded from the analysis. During the interview, the unexpected presence of the interviewee’s superior created external pressure, which likely influenced the responses and compromised their authenticity and reliability.^50^,^52^ The presentation of the KII and FGD data is compliant with the Consolidated criteria for Reporting Qualitative research (COREQ) guidelines.^53^

### Patient and public involvement statement

Patients or the public *were not* involved in the design, conduct, reporting or dissemination plans of our research.

## Results

### Sociodemographic characteristics of the participants

A total of 21 KIIs were conducted; 10 in Moatize district and 11 in Moma district (table 1). In addition, 35 caregivers participated in four FGDs, two in each district. Overall, there was a considerable overlap of the topics discussed during the KIIs and the FGDs, indicating a consistent pattern in the themes explored through both methods. Of note, only one interviewee refused to be recorded and suggested a group interview rather than an individual one. Hence, this particular interview transcript was excluded from the final analysis in order to maintain a high level of consistency of the methodology and analysis.

**Table 1:** Characteristics of participants from key informant interviews

Key informant interviews	Moatize (n=10)	Moma (n=11)	Total (n=21)
Number and gender of study participants in KIIs			
Male	6	8	14
Female	4	3	7
Average age in years (range)			
Male	50 (35–69)	41 (30–63)	45 (30–69)
Female	46 (36–52)	45 (28–63)	45 (28–63)
Level of education (as per highest degree obtained)			
Male			
Primary school (1–7 years)	0	0	0
Secondary school (8–12 years)	2	2	4
University (Bachelor or Master)	4	6	10
Female			
Primary school (1–7 years)	1	0	1
Secondary school (8–12 years)	0	1	1
University (bachelor or master)	3	2	5
Sector			
Community representatives	2	1	3
Government	4	4	8
Mining	0	1	1
NGO	4	4	8
Civil society	0	1	1

NGO, non-governmental organisation.

All key informants worked in the public or private sector and had at least 5 years of experience in working with adolescents. The key informants were government employees (n=8), who worked in NGOs (n=8) and the community (n=3), were civil society representatives (n=2) or in the mining sector (n=1) (table 1).

The two FGDs, comprised one group of female participants (n=18) and one group of male participants (n=17). With regard to education, the majority of participants (18/35) had completed primary school in both districts (n=9 in Moatize and n=9 in Moma), followed by illiterate participants (n=4 in Moma and n=4 in Moatize). Services and sales workers and skilled agricultural and fishery workers made up the majority of participants’ occupations (table 2).

**Table 2:** Characteristics of participants from focus group discussions

Focus group discussions (FGDs)	Moatize (2 FGDs with a total of 16 participants)	Moma (2 FGDs with a total of 19 participants)	Total (4 FGDs with a total of 35 participants)
Number of participants in FGDs			
Male	7	10	17
Female	9	9	18
Average age in years (and range)			
Male	56 (35–67)	44 (29–58)	49 (29–67)
Female	35 (30–60)	31 (20–43)	33 (20–60)
Total	44 (30–67)	38 (20–58)	41 (20–67)
Average number of years of school attended (and range)			
Male			
None	1	3	4
Primary school (1–7 years)	3	6	9
Secondary school (8–12 years)	3	1	4
Female			
None	1	3	4
Primary school (1–7 years)	4	5	9
Secondary school (8–12 years)	4	1	5

### Perceived health impacts and cross-cutting issues

Participants in this study reported various common health issues affecting adolescents in both study areas (figure 3). Overall, sexual and reproductive health issues were perceived to be the major health problems concerning adolescent health and well-being in the affected communities, with most of the participants specifically highlighting HIV among the STIs. This was followed by respiratory tract infections, diarrhoeal diseases and malaria (figure 3).

### Perceptions of sexual and reproductive health of adolescents in mining areas

Sexual and reproductive health emerged as a major concern in the two study sites, as revealed in the KIIs and FGDs. Most of the participants in Moatize district mentioned STIs and agreed that HIV/AIDS is a general health problem affecting adolescents in mining communities, followed by syphilis and gonorrhoea (figure 3). Moreover, unplanned or early pregnancies and abortions were also frequently mentioned.

These are the most common diseases in young people [referring to STIs such as HIV/AIDS]. Some get diseases, they hang out with men, girls get sick at home until they are about to die. They go to the hospital for analysis and it turns out that they already have the disease [referring to HIV]. (FGD with female caregivers of adolescents, Moatize).

Interviewees and the focus group participants associated significant and contextual aspects of adolescent sexual and reproductive health in impacted communities. Respondents perceived commercial sex work as the main driver contributing to the spread of HIV/AIDS and other STIs in mining areas. Of note, participants mentioned adolescent girls to be more vulnerable to the consequences of risky sexual behaviour in mining areas, as only adolescent girls were mentioned to be engaged in commercial sex work, along with other sexual activities, often starting at a very young age, around 13–14 years.

Participants in this study believed that due to limited resources and inadequate health education in mining areas, adolescents engaged in risky behaviours such as early sexual debut and unprotected sexual intercourse. These behaviours are perceived to affect adolescents’ sexual and reproductive health by increasing commercial sex work, unwanted pregnancy and HIV in the mining host communities. As an example, respondents reported that the lack of financial resources had drawn some girls into commercial sex work as a means of acquiring material goods and, at the same time, exposed them to violence in mining areas.

So poverty makes young girls accept anything. Especially for girls. Because it happened, there have been unwanted pregnancies, episodes of abuse, and these [referring to risky] behaviours. (KII S2, Moma).

Participants also stated that in mining communities, the increased lack of economic means and livelihood opportunities leads to adolescent girls being encouraged to get married earlier to alleviate the family financial shortages. This was perceived to be exacerbating pre-existing cultural beliefs encouraging early marriages. One participant stated that *“caregivers marry their daughters early because they could not afford to support them, and they see marriage as a way to reduce their financial burden*”. The lack of stable support systems and economic opportunities is perceived to further exacerbate their exposure to early sexual debut, transactional relationships and early marriages, as highlighted by participants in our study. Consequently, the multiple disadvantages and precariousness experienced by adolescents were perceived as important aspects affecting adolescents’ sexual and reproductive health.

Some have, some don’t have, but they claim poverty as a reason […] one of the ways to overcome poverty or to cover the house expenses, is to take their daughters to some men. The girl was already attending school at the fifth grade at the age of 14 but the father claimed lack of conditions to maintain or continue the studies. (KII S1, Moma).

Mining was generally perceived to influence sexual practices of adolescents negatively; yet, the key informant representing the mining sector believed that mining activities did not directly contribute to commercial sex work in the community. Instead, he attributed the phenomenon to location-specific characteristics of the district. In his view, commercial sex work among adolescents is more common in districts located along the coast, where poverty exacerbates the vulnerability of adolescent girls, many of whom are already heads of households or need to support their families.

Prostitution exists at any place. We are talking about the district itself. I am not saying that prostitution is linked to the mining company […]. You see the level of vulnerability of the girls, perhaps they need 50 Meticais [local currency] to meet a family need and despite being of a certain age [minors], the majority are already heads of their families. Because they lost caregivers, and were left to take care of their siblings. (KII S8, Moma).

The key informants and focus groups identified migration as another major driver of commercial sex work in mining areas, as it was perceived to increase the demand for it. Two factors with regard to migration were identified to be particularly important: (1) the influx of people to the districts and (2) the mining areas as hubs of opportunities. Participants perceive that the two districts form a mining area corridor characterised by high mobility, attracting and increasing the number of migrant workers. Respondents identified them as mostly young men separated from their families and having better financial resources. Participants recognised that the financial resources provided by migrant workers attract adolescent girls to engage in commercial sex work. At the same time, this influx of resources might disrupt the traditional values and prospects of the adolescents in these communities, as illustrated by the following quote:

Yah, they are young men, between the ages of 20, 30 and 35 and many far away from their families or their town […]. They are people with more financial means, enough to resolve some specific issues in the medium- or long-term and this makes them the kings of the land. So what do we expect from these communities that these people are inserted? (KII S5, Moatize).

Further, the mining communities were perceived by respondents to attract adolescent boys and girls due to the opportunities for work and trade, which exposes them to other risky behaviours, such as substance abuse (ie, alcohol and drugs). Participants from both districts echoed that mining areas have created conditions that contribute to increased substance among adolescents and school drop-outs.

Our adolescents in neighbourhood, many donʼt want to go to school, and spend nights drinking, taking drugs, they don’t do what adolescents should do, they are deviated. (FGD with male caregivers of adolescents, Moatize).

### Respiratory and water-borne diseases in adolescents living in mining communities

A specific concern regarding adolescent health identified by the study participants was related to increased air and water pollution, mainly due to dust in surrounding mining areas. These issues were mostly perceived by KII and FGD participants from Moatize district. Mining activities contributed to increased respiratory diseases in their communities, including tuberculosis, cough and influenza. Participants attributed the increase in respiratory diseases to the high levels of dust generated by mining companies in the communities. For example, a key informant who works in a health facility highlighted that the increased number of patients suffering from respiratory diseases is directly related to polluted air.

Cases of environmental pollution, we know very well that in Moatize, there is a lot of environmental pollution. And where there is a lot of environmental pollution, it is certain that lung diseases or respiratory tract diseases, for example issues such as pulmonary tuberculosis and bronchopneumonia, so bronchial asthma due to excessive irritation with coal dust these diseases can occur. There is still no concrete study on this but as a health technician I already know very well what happens in a polluted area where people breathe polluted air. (KII S5, Moatize).

Further, caregivers also expressed concerns about air pollution in the surrounding mining areas. The high level of pollution was perceived to be linked to the extraction methods employed in mining exploration, particularly explosions in open-pit coal mine in Moatize district. Most caregivers noted that respiratory diseases were not prevalent before the implementation of open-pit coal mining. In particular, the respondents expressed concern that children and adolescents were vulnerable to adverse effects of these mining activities. According to the respondents, air pollution was often perceived as ubiquitous, as illustrated by the following quote:

I would like to reinforce that many years ago, these types of diseases did not exist. Back then, when [name of previous mining company] was exploring coal, they were underground mines so the only people who could catch diseases would be those who were exposed to and working in the mining, now with the [name of the new mining company] the mines are open so all the dust comes out of the mines and goes into the houses, even the white flour [meal prepared with maize (locally called “xima”)] and coal dust are mixed into food [visibly affected by the dust], so that’s why I said there is more tuberculosis than in the past years. (FGD with male caregivers of adolescents, Moatize).

Also directly linked to the extraction methods (explosion), most respondents in Moatize district expressed their disappointment, given that mining companies are not carrying out any action to address the health of the communities. They stated that they were unaware of any mitigation plan from the mining companies to minimise the existing impacts in the short term or long term. However, we could not confront these perspectives, as the mining representatives’ insights in Moatize district were not captured in this study. Key informants and FGD participants emphasised the need for urgent community intervention programmes from mining companies to reduce morbidity, mortality and the health risks they are exposed to in the areas where mining companies operate. Participants perceive that this negligence by mining companies has led to a deterioration in the health of adolescents, as stated by one of the key informants.

There are explosives from one side to another and we don’t always have masks to protect ourselves and we end up catching these diseases. (KII S8, Moatize).

Our adolescent are directly affected, I said it well that the biggest problem is the dust, so our young people are the ones who are always exposed, they don’t always wear masks, even the mask we use now because of COVID-19. Just to know that the mining companies have been here for more than 10 years, so yeah, the biggest problem here is the dust, they inhale a lot of dust. (KII S6, Moatize).

Similar to findings related to respiratory diseases, participants in Moma district agreed that the mining excavation leads to big holes and further intensifies the spread of dust through the communities and into the water bodies. Key informants and FGD participants in Moma district explained that the open holes left by mining companies are contributing to an increase in water-borne problems, such as diarrhoeal diseases. Participants described specific situations in which adolescents are exposed, highlighting the constant threat to health and well-being. One respondent gave an example of the increase in reported cases of diarrhoeal diseases in recent years, which they also associated with the negligence of mining projects operating within their communities.

### Rising malaria concerns in mining sites

There was consensus among participants in both study sites that malaria is a major contributor to morbidity and mortality in the communities, mentioned by more than a third of the key informants (9/21) and most participants in the FGDs (20/35). In comparison to previous health issues mentioned, there was a general agreement that malaria is an important problem in the community, exacerbated by the presence of mining companies and how they operate. The increase in malaria was perceived to mainly affect the most vulnerable groups, such as children and adolescents.

Among these diseases that we talk about here, the ones that most affect adolescents are two diseases. The first is malaria […], since we received the company here [referring to mining company], mosquitoes bite all the time. So we think these mosquitoes come from the company. (FGD with male caregivers of adolescents, Moma).

Participants also reported the existence of big holes created by mining activities that serve as breeding sites for mosquitoes and contribute to the increased prevalence of malaria in these mining communities. In their view, increased exposure to mosquitoes in both neighbourhoods and residences augments the risk of being bitten, thereby facilitating the spread of mosquito-borne diseases.

### Stakeholders’ views on adolescent health and challenges in mining communities

Participants across various levels of the health system reported limited capacity of the facilities and fully equipped and functioning health services to provide the best quality healthcare and meet the health needs of adolescents. In addition, the long distances to reach the health facilities and lack of available medicines to treat the aforementioned health conditions were perceived by most key informants as major obstacles for improving adolescents’ overall health status and seeking care in mining communities.

We are looking for medicine, but when we arrive at the hospital, we cannot find medicine to combat these diseases that affect adolescents and they end up coming back anyway, this way the disease gets worse. If they only go with a headache, when they arrive at the hospital they say here they only have paracetamol […]. When we go we canʼt find any medicines, and this hospital is far from us. (FGD with male caregivers of adolescents, Moma).

The majority of caregivers in both Moma and Moatize districts raised similar concerns, highlighting the insufficient and inappropriate availability of integrated adolescent-friendly health services in these mining communities. Consequently, adolescents are reported to often resort to seeking alternative treatments, such as traditional healers. In addition, caregivers mentioned stigma associated with STIs as one of the main barriers for adolescents to seek care at health facilities.

For me, it is visible to see this stigma, the shame, it has caused candidiasis, syphilis or gonorrhoea and they start to see that you are sick. But if they go to a traditional healer and talk to somebody, they can hide and not be recognised. (FGD with male caregivers of adolescents, Moatize).

### Opportunities for adolescent health in mining communities

**Figure 4:**
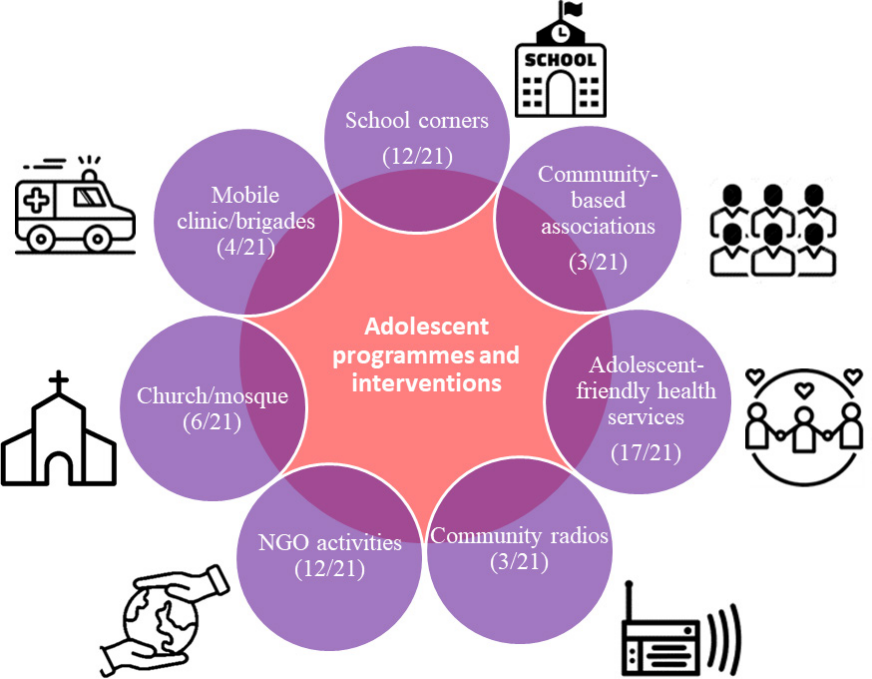
Available interventions and programmes addressing adolescent health, Moma and Moatize districts, Mozambique, 2022. NGO, non-governmental organisation.

In our study, key informants and caregivers also had various opinions about the health needs of adolescents in the mining communities. They identified existing local programmes and intervention initiatives implemented by different sectors to address the negative health outcomes. According to them, different programmes have been implemented in health facilities, schools and communities to target adolescents in mining areas. The programmes are managed by the government, NGOs and actively engaged local community members (figure 4). Hence, most key informants identified adolescent and youth-friendly health services as the most appropriate intervention for providing specific services for adolescent care (17/21). In addition, school corners (12/21), NGO activities in the community and hospital (12/21), religious groups (church/mosque) (6/21), mobile brigades (4/21), community radio (3/21) and community-based associations (3/21) were also mentioned by participants as available within the community for adolescents.

Contrary to the general negative perceptions, key informants and FGD participants from both districts mentioned recent efforts by one of the mining companies in Moma district to provide support to adolescents in the surrounding areas. Participants pointed to the positive impacts of mining companies when referring to infrastructure investments, including the rehabilitation and the provision of supplies for health facilities, the acquisition of ambulances and the construction of schools in the neighbourhood. Furthermore, the key informant representing the mining company admitted that they received several requests from community members. However, the company was unable to employ adolescents due to the legal restriction on age and the lack of necessary skills to work in the mine. Instead, the respondent highlighted scholarship programmes and investment in sports that the company provides for adolescents in the affected communities.

We have received several requests, but on the other hand, these young people do not have training. They want to work, but they don’t have any specific training to do that. For example, we have this issue of scholarships that we offer, also depending on the needs, not all communities have people with certain qualifications, because people want to receive these things in an easy way […] anyone who has availability and who is in a position to do so, the scholarships are open to everyone, looking at those who are available or have a vocation to undertake some training. (KII S8, Moma).

Despite the cited instances of support from the mining company for adolescent education, the construction of schools and the provision of school supplies, key informants and caregivers in Moma district emphasised gender issues around the beneficiaries of these initiatives. Participants highlighted the lack of equitable distribution of resources and opportunities for boys. They recommended equitable support in order to benefit all adolescents in the surrounding communities, not being limited by gender or geographical location.

Especially in these scholarships they want more these girls. It’s for students who are here [refering to neighbourhood name close to the mine sites]. (KII S3, Moma).

Participants reported unemployment as a frustration for adolescents living in mining host communities. They expressed concern about the lack of opportunities and support for adolescent boys to work for the mining companies. According to their perspective, adolescents are not viewed as a potential future workforce worthy of investment, despite their capability to contribute to the transformation and improvement of the economic situation in their community. This perceived lack of investment was seen to increase illegal activities, such as theft, in the communities.

They could help with anything, so adolescents could have a job. They should at least offer courses, because other communities we have heard that such a course is being offered. Maybe if they had a certificate they could be called too. Because young people have nothing to do, no other profession, just stealing, but if they had another occupation, they can say that I took this course and they could benefit. (FGD with female caregivers of adolescents, Moatize).

In addition, caregivers revealed that they had earnestly sought support from these companies, particularly through letters requesting assistance for adolescent initiatives. Despite their efforts, they did not receive positive feedback. Key informants from the health sector saw this experience in Moatize as an indication of the current lack of a supportive environment and collaborative efforts among stakeholders and mining companies.

I’m saying that I tried to send a letter, I deliver the letter saying that we have our children that are who might be able to work in the company. We heard they took other people and adolescents end up like this [refers to local concerns about the lack of employment opportunities for young people in mining areas]. Now they don’t want us inside [refers to restrictions placed on local communities regarding access to the mining concession area], but these companies are located in our territory. (FGD with male caregivers, Moatize).

While the mining representative primarily reported on support, listing all of the community-level interventions, he also acknowledged the challenges in balancing community expectations and shared decision-making among mining companies and different community subgroups within the impacted communities. He expressed the difficulty of addressing these communities’ needs, given that mining is not a philanthropic activity.

They [community members] say we want this and requested this, but there are procedures that we have to use to ensure that there is easy communication and understanding so that young people, and people from the communities understand the role of the company, because when there is no such knowledge, people always end up living with expectations and end up getting frustrated because sometimes they can have expectations that are not planned, and it is not easy to respond to something that is not previously planned. (KII S8, Moma).

Moreover, key informants and caregivers also reported their disappointment about the mining company’s commitments, particularly in support of an adolescent health agenda. As an example, one of the key informants representing the school health officer in Moatize district shared the challenges encountered while attempting to engage with mining companies.

We are doing our part as health sector, but we need support because we have limitations, we have already written letters asking for this, this, and this. But what happens, the letters are not answered. I don’t want to cite examples and cite sources, but we already wrote letters. For example, I wrote several letters myself on behalf of the school health programme, the letters have negative responses, we do not provide funds for this, at the moment we have nothing. (KII S10, Moatize).

Key informants also acknowledged the higher pressure versus limited capacity and resources from the health sector to cope with all aspects related to adolescent health and well-being in mining areas. The mining representative referred to the important role of the government in improving the well-being of their communities and recognised the contribution of mining companies in providing the necessary support for community-based interventions.

In order to accommodate and tackle these challenges, key informants suggested an intersectoral and multisectoral collaboration among relevant actors to address the health needs of adolescents in surrounding mining settings. For example, key informants in both sites highlighted the nature of the government-industry relationship as an important opportunity to incentivise mining companies to take immediate actions to support an adolescent health agenda in these communities.

It is the government itself that should get in touch with mining companies to help us for the health and well-being of our adolescents and young people. Hmm, this issue of increasing the number of schools, equip the health facilities, support us with medical-surgical material there in the hospitals, we have a lot of shortages, but they should give us a hand in this part of surgical materials. (KII S8, Moatize).

## Discussion

This qualitative study aimed to explore the perspective of a range of stakeholders and caregivers of adolescents on mining-related effects on adolescents’ health and well-being in two rural industrial mining areas in Mozambique. Combined findings from the KIIs and FGDs identified STIs, malaria, respiratory tract infections and diarrhoeal diseases as the four main health concerns affecting the health of adolescents in the study sites. The perception that the identified main health concerns are exacerbated through the presence of the mining operations was shared among all study participants. Although investment in public infrastructure, such as schools and health facilities, was recognised to be part of mining companies’ efforts in mitigating potential negative externalities, the interviewees and FGD participants perceive a lack in measures that specifically address health needs and risks in the adolescent age group in affected communities.

### STIs among adolescents in mining areas

Our findings suggest that sexual and reproductive health, particularly the increase in STIs, is a major concern affecting adolescent health and well-being in communities affected by extractive industries. The complex web of pathways associated with a high HIV burden has previously been identified as a major health concern in mining areas in SSA.^54^,^56^ Contributing risk factors specific to the adolescent age group include migration, economic marginalisation, early sexual debut, unprotected sexual activities, transactional sex and various forms of violence.^8 56 57^ In line with the findings of our study, the younger generation engaging in commercial sex work in communities hosting mining projects was reported to account for increased HIV prevalence in mining areas in Ghana,^56^ Mozambique and South Africa.^58 59^ A significant concern in Mozambique, one of the countries with the highest HIV prevalence in the world, is the alarming proportion of commercial sex workers who are adolescent girls^60^ and minors.^61^ This issue is particularly pronounced in rural areas, where limited access to education, healthcare and economic opportunities exacerbates vulnerabilities.^62^ Despite the growing body of evidence on the critical role the adolescent age group plays in elevated HIV transmission observed in communities affected by mining projects, our study confirms that there is a need to strengthen interventions that prevent adolescents from engaging in risky sexual behaviours, which has the potential to substantially contribute to the prevention of STIs in communities affected by extractive industries.^62^,^65^

Indeed, adolescents are often overlooked in HIV care, benefitting less from established service delivery models compared with other groups.^58^ Furthermore, given their importance as potential future worker force, particularly in SSA, addressing HIV and ensuring their overall well-being becomes even more imperative for communities impacted by mining because of the important role of this sector in shaping societal productivity and development.^59^ To date, only a few examples from SSA of promising interventions to address gender risks and vulnerabilities among adolescents in mining sites are available.^33^ A study in Zambia found that voluntary HIV counselling and testing in young people can make an important contribution to STI/HIV prevention in mining areas.^66^ In rural Mozambique, a study evaluating the performance of a direct-assisted oral HIV self-testing intervention among adolescents showed encouraging results by concluding that over 80% of adolescents preferred direct-assisted oral HIV self-testing at health centres.^67^ Both examples indicate that for similar developed rural settings, redesigning and promoting adolescent integrated care services can make an important contribution to preventing STI transmission in mining sites.^17^

### Increased malaria in mining communities

In our study, key informants and caregivers in Moma district associated mining with an increased malaria prevalence in surrounding communities. The large water pits created by mining companies were perceived to intensify the cases due to their operation. Similar to our results, an increase in malaria cases has been associated with mining activities, particularly in areas with surface-level mines due to the numerous pits dug for mining activities.^68^ This was confirmed in a recent study about extractive industries’ impact in the Democratic Republic of the Congo, showing that malaria prevalence was elevated among individuals exposed to mining.^69^ Similarly, a study conducted in Côte d’Ivoire found that malaria prevalence in mining settings remained stable, while participants had limited knowledge about malaria transmission.^70^ Another study conducted in gold-mining areas in Latin America evidenced an increase in malaria in mining regions and concluded that lack of malaria control activities, together with high migration and proliferation of mosquito breeding sites, contributes to malaria in gold-mining regions.^71^

While significant investments are directed towards controlling and eliminating malaria in SSA, its elevated prevalence rate in many mining areas, as opposed to non-mining areas, is a cause for concern. Our observations underscore the pressing need to deepen the epidemiology and risk factors involved in malaria transmission among population sub-groups in mining settings in SSA.^56^ Our study was conducted in northern and central regions of Mozambique, which are known as the most endemic settings for malaria transmission.^57^ This aligns with the recent WHO report, highlighting that malaria remains the primary cause of morbidity and mortality in SSA, with Mozambique among the four countries with the highest rates of malaria clinical cases and deaths worldwide.^53^

Malaria is a disease with a major impact on the social and economic development of a community. In tropical countries with a high burden of malaria, the potential health impact of extractive projects in exacerbating sanitation issues is of particular concern.^72^ Hence, our findings highlighted the importance of designing interventions that are tailored to particular social-ecological settings, such as mining areas in SSA. In addition, there is a need for monitoring potential project-related impacts over time, particularly in response to changes in ecological and sanitation systems.^70^ Thus, collaboration between the mining sector and national control programmes might effectively reduce the risk of vector-borne diseases in settings with high prevalence in SSA. However, further research would be valuable in exploring whether private-public partnerships could enhance malaria prevention efforts in these regions.^69^

### Strengthening collaboration and engaging key stakeholders in mining areas

Key informants and caregivers identified challenges within existing health programmes and interventions aimed at addressing the health needs of adolescents affected by mining sites. In addition, key informants revealed weak involvement and commitment of mining companies in supporting an adolescent health agenda, which is in line with previous studies that have demonstrated mining companies’ reluctance to address health issues in the communities in which they operate.^35 73^ For example, while early marriage is not unique to mining communities in Mozambique, the economic disparities and livelihood opportunities created by the presence of industrial mining are perceived to enhance existing social dynamics, including marriage practices among adolescents.^32 74^ Given the gaps in this domain, especially in mining settings in low-HDI countries, understanding the various stakeholders’ views, including the mining sector, is crucial as many project-induced health challenges need to be mitigated through multisectoral action.^75^ This claim is substantiated by a recent literature review, highlighting the importance of interventions that understand and support adolescents in mining communities by adopting multilevel, multisectoral and multistakeholder approaches, taking into account gender risks and vulnerabilities.^76^ A better understanding of the health of adolescents in these resource-constrained communities, marked by many challenges, including conflicts and tensions between communities and mining companies, power imbalances and intersecting economic, social, environmental and health issues, is crucial to inform appropriate policies that support their well-being.^77^ While much of the focus has been on adult perceptions, the perspectives of adolescents themselves on the determinants of their well-being in resource extraction areas are likely to provide valuable insights, as adolescent literature demonstrated multiple needs extending beyond their (physical) health.^39^ Additionally, differentiating and stratifying these perspectives across vulnerable subgroups is crucial for advancing health equity and ensuring targeted, effective interventions.^78^ Evidence in the literature points to potential positive health impacts on affected communities in SSA. Studies have shown that mining companies that integrate CSR initiatives that support education, vocational training and economic empowerment programmes for adolescent girls and their families have the potential to mitigate the economic vulnerabilities that contribute to early marriages.^79^ By fostering a multisectoral approach, stakeholders can create a more protective environment for adolescents in mining communities, ensuring their rights, health and well-being are safeguarded.^80 81^ Few cases were found in SSA where the mining sector CSR actions have contributed to improving sexual and reproductive health and education among adolescents, for example in Zambia, the mining company owns and operates schools as part of its contribution to social sustainability. The school provides quality primary education to over 1000 children, both mine and non-mine employees, and it stands out as one of the best schools in Zambia.^82^ This involves understanding the local policy context for multisectoral action for adolescent health and supporting governance approaches within and beyond the health sector to initiate, sustain and lead the necessary changes, with platforms and teams across sectors supported by established coordination mechanisms.^62^ For instance, school corners in Mozambique play a crucial role in promoting a safe and supportive educational environment, addressing students’ concerns and contributing to their overall well-being. This programme is known for its peer support approach, involving adolescent counsellors and young people, who aim to respond to questions simply and ‘youthful’. While school corners are the most prominent example of a functioning adolescent health programme in the country, resources for developing similar programmes for vulnerable groups, such as adolescents residing and working in affected communities, remain scarce.^83 84^ Therefore, health interventions that adopt a more holistic approach and are tailored for adolescents represent a valuable opportunity to improve teacher-parent-child communication and protect them from making unhealthy choices.^65^ Additionally, targeted interventions focusing on education, economic empowerment and environmental safety are essential to mitigate adolescent risks, as well as other potential hazards, such as drowning and injuries, which require further exploration. Future research should investigate the broader impact of these challenges on adolescents’ health to inform the development of effective policies and interventions.^39^

### Opportunities for intersectoral collaboration

SDG 3 presents a unique opportunity to emphasise adolescents’ health needs and reinforce effective multisectoral action, including public-private partnerships.^62^ As long as the extractive industry sector is considered central for development and economic growth in SSA, investments in health and well-being are an opportunity to strengthen sustainable collaboration among the public and private sectors in resource-rich regions.^66 85^ Key informants in this study highlighted the importance of government-industry collaboration to provide an opportunity to expand the involvement of mining companies in committing to and supporting the adolescent health agenda in affected communities. This can be promoted by establishing platforms that are jointly used and maintained by public and private actors, such as the Resource Information Dashboard.^86^ Moreover, prospective approaches that aim to promote multisectoral action for health, such as health impact assessment (HIA), should be applied in the licensing process of extractive industry projects.^87 88^ Indeed, HIA, which follows the guiding principles of participation, equity and equality, holds potential to safeguard and promote health and well-being of adolescents in communities affected by large infrastructure projects.^28^ In countries like Mozambique, where HIA is not routinely implemented, government and private sector policies and guidelines to address the needs of adolescents in mining areas remain of importance.^28^ However, a better description and further development to specifically address adolescent health and well-being would be desirable, alongside guidelines, frameworks and practical tools tailored to adolescents.^38^ Hence, more qualitative and participatory HIA methods are needed to understand those issues better, especially understanding those that specifically engage adolescents and grasp insights behind health in capturing adolescent health outcomes, particularly among vulnerable subgroups such as adolescent girls.^39^

### Strengths and limitations

The strengths of the study rely on its capability to include in-depth insights and perspectives of those who care for, make decisions on behalf of and serve adolescents (caregivers and multiple key informants from government staff, the private sector, NGOs and civil society), providing contextually relevant findings to inform policy and practices. Additionally, the study successfully captured the insights from two types of mining industries, providing a comprehensive understanding of their distinct challenges and perceived environmental and socioeconomic impacts. The study, however, has study has several limitations that are offered for discussion. First, the findings reflect caregivers’ and different stakeholders’ perceptions and may not necessarily align with the actual experiences and perspectives of adolescents in mining areas. While the experiences of adolescents are presented elsewhere,^39^ the current findings complement the broader understanding of the challenges and dynamics faced by this group in these mining communities. Second, we only included stakeholders in our study who worked in institutions that aimed to support adolescents in both study sites. Working in rural settings, we are likely to have missed informal groups and individuals trusted by adolescents who may also provide healthcare, such as traditional healers, matrons and religious leaders. Third, the study only focused on two specific mining areas in one country and at a single point in time, which may not reflect long-term trends or changes across different geographic locations. Hence, the findings may not be generalisable to all mining areas across SSA.

By focusing on specific groups, the study may not fully capture the complexity of the issue, as it does not account for broader external factors, such as national policies or economic conditions, which could influence the experiences of caregivers and stakeholders. We acknowledge the potential selection bias in our sample, particularly regarding under-represented perspectives due to sensitivities around a local employer (mining company). Thus, we recognise that some viewpoints may still be under-represented and suggest future research using ethnographic methods or longitudinal studies to address this. Finally, conclusions regarding the perceptions of mining should be carefully interpreted; given that the majority of mining representatives were not accessible at both study sites, our findings are limited to the perspective of one representative, which limits the depth and richness of the analysis. However, the one interview that we managed to conduct with a mining representative did provide relevant insights in the perspective of the mining sector.

## Conclusion

Our study sheds light on the multifaceted challenges adolescents residing in mining communities in SSA face. The consensus among different stakeholders and caregivers of adolescents highlighted the perceived adverse impacts of the mining environment and the dynamics it generates on adolescent (risky) behaviours, the natural environment, the socioeconomic status and the health of adolescents residing in surrounding communities. The lack of priority given to effective interventions that target adolescent health outcomes and safeguard their overall well-being underscores the importance of more intersectoral collaboration to adequately address adolescent health and well-being in mining areas through age group-specific interventions. Additionally, targeted interventions focusing on education, economic empowerment and environmental safety are essential to mitigate the identified risks, as well as other potential hazards, such as drowning and injuries, which require further exploration. Future research should investigate the broader impact of these challenges on adolescents‘ health to inform the development of effective policies and interventions. Safeguarding and promoting health and well-being of adolescents in mining areas is not only in line with the 2030 Agenda for Sustainable Development and its aspiration to ‘leave no one behind’ but will also benefit companies themselves since adolescents will be part of their potential future local workforce.

## Supplementary material

10.1136/bmjopen-2024-088207 online supplemental file 1

10.1136/bmjopen-2024-088207 online supplemental file 2

10.1136/bmjopen-2024-088207 online supplemental file 3

## Data Availability

Data sharing not applicable as no datasets were generated and/or analysed for this study. Data are available upon reasonable request. All data relevant to the study are included in the article or uploaded as supplementary information.
