# Supplementary material for: Understanding stakeholders’ perceptions of the impact of extractive industries on adolescent health and well-being in Mozambique: a qualitative study
Source: BMJ Open. 2025 Jun 6;15(6):e088207. doi: 10.1136/bmjopen-2024-088207 (PMC12161353; doi:10.1136/bmjopen-2024-088207)
Supplement: online supplemental file 1 [file bmjopen-15-6-s001.DOC]

|  | | |
| --- | --- | --- |
| ***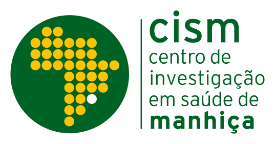*** | **Manhiça Health Research Centre (CISM**  Adolescent health well-being in the context of natural resource extraction projects  KEY INFORMANT INTERVIEW GUIDE  PEE_CS_Adolescent study_QA_001_A05_v01_PT  Data  __/___/____  Version 1, February 2022  Versão 1, Setembro 20201 | 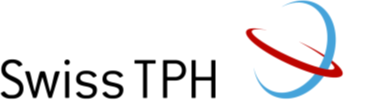 |

My name is ___________________ from the Manhiça Health Research Center, and I would like to welcome you to this interview. I will be the facilitator of this interview, and with me is Mr./Ms. _______________ also from the Manhiça Health Research Center, who will take notes and record the interview with your permission.

**Purpose**

This interview aims to collect data that will help us understand the impact of mining on the health and well-being of adolescents in Mozambique. As members living or working in this community, we ask you to share with us your knowledge and experience regarding the community before and after the implementation of mining companies in this district. Your participation and contribution are very important; thus, you were invited to participate in this interview to provide information that will help us learn more about the health and well-being of adolescents living and working in this community. The duration of this interview is expected to be between 30 and 60 minutes.

Do you have any questions or comments before we proceed? (responses)

I will ask your permission ad now turn on the recorder so we can accurately remember your experiences and opinions. (Turn on the recorder.)

Instructions for the note-taker: Fill in the participant’s profile information before starting the interview. The participants’ informed consent forms should be completed in advance. Below is a study summary that the interviewer should use to ensure that participants understand the information in the consent form.

# PARTICIPANT CHARACTERISTICS

| Ref. **-\|__\|__\|__\|- -\|__\|__\|-\|**  Site Participant Profile | | | | |
| --- | --- | --- | --- | --- |
| **Date**: \|___\|___\|/\|___\|___\|/\|___\|___\| | | **Location**: ____________________________________________________________ | | |
| **Sex: Feminine Masculine** | | **Age do p** **Participant’s Age: \|**: \|__\|__\| | | |
| **Level of Education:** | | **Occupation:** | | |
| **Start Time:** \|__\|__\|:\|__\|__\| | | **End Time:** \|__\|__\|:\|__\|__\| | | |
| **Language(s) spoken:**_________________________________________________________________________ | | | | |
| **Result of the interview** | **Recorded**  **Not recorded**  **Reason: ______________________________________**  **Complete**  **Interrupted**  **Reason: ______________________________________** | | | **☐ Unable to complete**  **To be completed on (date):**  **_______________________** |
| **Interviewer Initials:** \|__\|__\|__\| | | |  | |

|  | **Topic** | **Questions** |
| --- | --- | --- |
| **1** | Institutions working with Adolescent Health | ***   - Can you tell me a bit about your work? (Probe: type of institution and focus) - • Have you worked with adolescents? (Probe: What ages? What type of work? What areas?) - • Have you cooperated with the Government? Or with other organizations working in this area? - • What constraints have you faced in your work with adolescents? Why? |
| **2** | Knowledge about Community Diseases | - Could you tell me about the most common diseases in this community? - - Why do these diseases exist? What are the causes? - - Which ones affect adolescents the most? Why? - - What has been done to safeguard adolescent health? |
| **3** | Health-seeking Behavior and Health Maintenance | - What health options exist for adolescents? - • What type of health facilities or care do adolescents seek when they need health services? - • Where do adolescents in this community seek information to protect or improve their health? - • How has your institution contributed to improving adolescent health? |
| **4** | Barriers to Seeking and Accessing Treatment | - Have you observed a time when diseases increased in the community? What do you think were the reasons? - • What barriers or reasons might prevent an adolescent from seeking treatment when feeling unwell? - • Which group has benefited more from healthcare – boys or girls – and why? - • What type of health facility do you think is better for adolescents? (Probe: aspects they prefer) - • How could health services for adolescents be improved? |
| **6** | Impact of Mining | - In your opinion, is there any activity that places adolescents at higher risk of contracting diseases? - • Has the presence of a mining company contributed to improving adolescent health? Why? - • How are the mentioned diseases related to mining? - • How and why has it directly affected adolescents? - • Are there any benefits for adolescents from having a mining company in the district? - • What could be done to help improve adolescent health in this community? Who should take action? What role should each sector/actor have? |
| **7** | Final Questions | - Do you have any questions? - • Would you like to add anything else? |

**Instructions for the Moderator: Thank you note**

Thank you for your participation. This was a very successful interview.

Your input will be very valuable for the study.

If you are dissatisfied with anything or wish to file a complaint, you may tell me or contact the study coordinators listed in your consent form.

Please remember that all comments made during this interview will be kept anonymous.

**OBSERVATIONS:**

_______________________________________________________________________________________________________________________________________________________________________________________________________________________________________________________________________________________________________________________________________________________________________________________________________________________________________________________________________________________________________________________________________________________________________________________________________________________________________________________________________________________________________________________________________________________________________________________________________________________________________________________________________________________________________________________________________________________________________________________________________________________________________________________________________________________________________________________________________________________________________________________________________________________________________________________________________________________________________________________________________
