# Supplementary material for: Understanding stakeholders’ perceptions of the impact of extractive industries on adolescent health and well-being in Mozambique: a qualitative study
Source: BMJ Open. 2025 Jun 6;15(6):e088207. doi: 10.1136/bmjopen-2024-088207 (PMC12161353; doi:10.1136/bmjopen-2024-088207)
Supplement: online supplemental file 2 [file bmjopen-15-6-s002.docx]

|  | | |
| --- | --- | --- |
| ***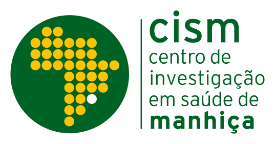*** | **Manhiça Health Research Centre (CISM**  Adolescent health well-being in the context of natural resource extraction projects  FOCUS GROUP DISCUSSION  Caregivers  PEE_CS_Adolescent study_QA_001_A03_v01_PT  Version 1, February 2022  Versão 1, Setembro 20201 | 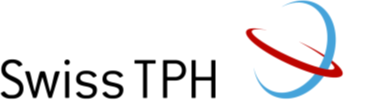 |

My name is ___________________ from the Manhiça Health Research Centre (CISM), and I would like to welcome you to this discussion. I will be the facilitator of this Focus Group Discussion (FGD) and with me is Mr./Ms. _______________ also from CISM, who will be taking notes and recording this discussion, with your permission.

**Purpose**

The objective of this Focus Group Discussion (FGD) is to collect data that will help us understand the health and well-being of adolescents in areas affected by natural resource extraction projects.

As caregivers and guardians of adolescents living in this community, many of whom have experienced life before and after the mining project implementation, your participation and insights are extremely important.

You were invited to participate in this FGD because we believe you can provide valuable information about the health and well-being of adolescents living and working in this community. This discussion is expected to last between 60 and 90 minutes.

**Rules**

We will ask your permission to record this FGD to ensure that none of your responses are lost. My colleague will also be taking notes during the discussion.

All recorded information will be kept confidential and no names will be associated with the information shared.

You are free to choose not to answer any question and may withdraw from the discussion at any time.

To promote group cohesion and ensure everyone has an equal opportunity to speak, we kindly ask that you follow these rules:

The most important rule is that only one person should speak at a time. It may be tempting to interrupt, but please wait until the speaker has finished. Everyone will have a chance to respond if they wish;

There are no right or wrong answers in this discussion;

You do not need to speak in any specific order;

When you would like to contribute, simply raise your hand or indicate it verbally. Since there are many participants, it is important that I hear from everyone;

You do not have to agree with the opinions of others. All viewpoints are respected.

Do you have any questions or comments before we begin? (Wait for responses.)

I will now turn on the recorder so that we can accurately capture your experiences and opinions. (Turn on the recorder.)

With your permission, we will start the discussion.

*Instructions for the Facilitator: Allow enough time for participants to think before answering. Use probing and follow-up questions to ensure all aspects are explored, but move forward when you begin to hear repeated answers.*

*Instructions for the Note-taker: Fill in participant demographic profiles before the discussion starts. Ensure that informed consent forms are completed beforehand. The facilitator should summarize and make sure participants understand the information presented in the assent form.*

# PARTICIPANT DEMOGRAPHIC INFORMATION

| **Participant ID (number and initials)** | **Age** | **Sex** | **Marital Status** | **Education** | **Occupation** | **Religion** | **Age of Adolescent Child** | **How long have you lived in this community?** |
| --- | --- | --- | --- | --- | --- | --- | --- | --- |
| **1** |  |  |  |  |  |  |  |  |
| **2** |  |  |  |  |  |  |  |  |
| **3** |  |  |  |  |  |  |  |  |
| **4** |  |  |  |  |  |  |  |  |
| **5** |  |  |  |  |  |  |  |  |
| **6** |  |  |  |  |  |  |  |  |
| **7** |  |  |  |  |  |  |  |  |
| **8** |  |  |  |  |  |  |  |  |
|  |  |  |  |  |  |  |  |  |

**GENERAL FGD INFORMATION**

| Ref. **-\|__\|__\|__\|- DGF -\|__\|__\|-\|__\|__\|**  Site Target group Number | | | | |
| --- | --- | --- | --- | --- |
| **Date**: \|___\|___\|/\|___\|___\|/\|___\|___\| | | **Location**: ____________________________________________________________ | | |
| **Initial number of participants: \|__\|__\|** | | **Número final de participantes**: \|__\|__\| | | |
| **Start Time:** \|__\|__\|:\|__\|__\| | | **End Time:** \|__\|__\|:\|__\|__\| | | |
| **Language (s) spoken:**_________________________________________________________________________ | | | | |
| **Brief description of participants:** | | | | |
| **Result of Focus group discussion** | **Recorded**  **Not recorded**  **Reason: ______________________________________**  **Completed**  **Interrupted**  **Reason: ______________________________________** | | | **If incomplete, to be completed on (date):**  **_______________________** |
| **If incomplete, to be completed on (date)** \|__\|__\|__\| | | | **Facilitator initials:**  \|__\|__\|__\| | |

|  | **TOPIC GUIDE** | **Questions** |
| --- | --- | --- |
| **1** | Leisure Activities | **Start with an icebreaker*  *- What activities do adolescents engage in during their free time in this community?*  *- What places do they frequent?*  *- What types of activities are most common? Why?* |
| **2** | Knowledge of Diseases | - Could you tell me about the most common illnesses in this community? - - Why do these illnesses exist? What are their causes? - - Who is most affected and why? - - Which illnesses most affect adolescents and why? |
| **3** | Health-seeking and Maintenance Behaviors | - What types of healthcare services do adolescents seek when they need medical care? - - What symptoms prompt them to seek care? - - Where do adolescents seek information to protect or improve their health? - - What type of information do they receive and why do they choose those sources? - - Are these sources trusted? - - What healthcare options exist for adolescents in this community? - - How does it differ from when you were younger? |
| **4** | Barriers to Healthcare Access | - - Have you observed periods when diseases increased in the community? What were the reasons? - - What barriers prevent adolescents from seeking treatment? - - How do adolescents overcome these barriers? Who supports them? - - Which group benefits most in terms of healthcare (boys or girls) and why? - - Which health facilities are best for adolescents and why? - - What services would you like improved to better meet adolescents’ needs? - - How could adolescent health services be improved in this community? |
| **5** | Impacto da mineração | - - In your opinion, are there activities in the district that put adolescents at greater risk of illness? - - Has the presence of a mining company improved adolescent health? Why or why not? - - How are mining-related illnesses linked to adolescents? - - Are there any direct benefits for adolescents from the mining company? - - What could be done to improve adolescent health in this community? Who should be responsible? What should the role of each actor/sector be? |
| **7** | Final Questions | - Do you have any questions? - - Would you like to add anything else? |

**Instructions for the Moderator: Closing**

- Thank you for your participation. This has been a very successful discussion.
- Your opinions will be very valuable for this study.
- If you are dissatisfied with anything or would like to make a complaint, you can talk to me or contact the study supervisors listed on your consent form.
- I remind you that all comments made during this discussion will remain anonymous.

**OBSERVATIONS:**

_______________________________________________________________________________________________________________________________________________________________________________________________________________________________________________________________________________________________________________________________________________________________________________________________________________________________________________________________________________________________________________________________________________________________________________________________________________________________________________________________________________________________________________________________________________________________________________________________________________________________________________________________________________________________________________________________________________________________________________________________________________________________________________________________________________________________________________________________________________________________________________________________________________________________________________________________________________________________________________________________________
