# Supplementary figures and images for: Understanding stakeholders’ perceptions of the impact of extractive industries on adolescent health and well-being in Mozambique: a qualitative study
Source: BMJ Open. 2025 Jun 6;15(6):e088207. doi: 10.1136/bmjopen-2024-088207 (PMC12161353; doi:10.1136/bmjopen-2024-088207)

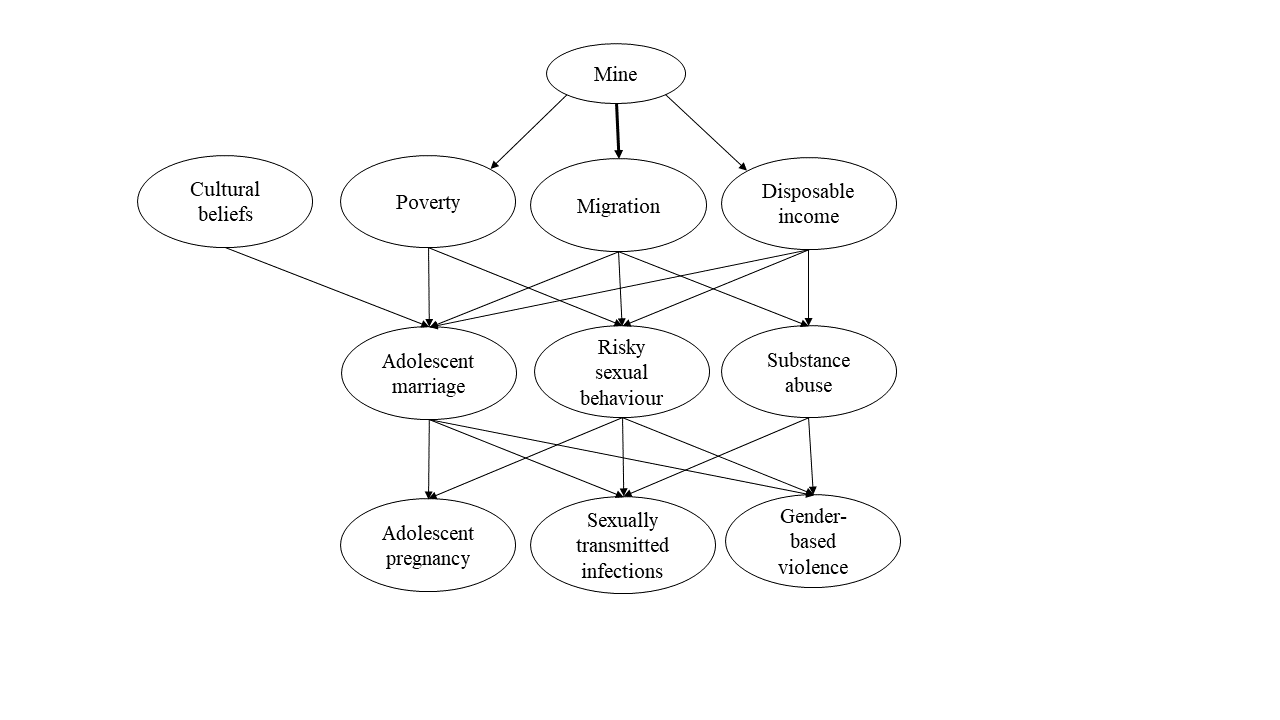

Supplement: online supplemental file 3 [file bmjopen-15-6-s003.png]
