# Supplementary material for: Understanding stakeholders’ perceptions of the impact of extractive industries on adolescent health and well-being in Mozambique: a qualitative study
Source: BMJ Open. 2025 Jun 6;15(6):e088207. doi: 10.1136/bmjopen-2024-088207 (PMC12161353; doi:10.1136/bmjopen-2024-088207)
Supplement: Supplementary file 4 [file bmjopen-2024-088207.reviewer_comments.pdf]

PEER REVIEW HISTORY

BMJ Open publishes all reviews undertaken for accepted manuscripts. Reviewers are asked to complete a checklist review form and are provided with free text boxes to elaborate on their assessment. These free text comments are reproduced below.

ARTICLE DETAILS

Title (Provisional)

Understanding stakeholders' perceptions of the impact of extractive Industries on adolescent well-Being in Mozambique: a qualitative study

Authors

Cambaco, Olga; Brugger, Curdin; Munguambe, Khátia Rebeca; Utzinger, Jürg; Winkler, Mirko S; Galvão, Christoff; Knoblauch, Astrid Madeleine

---

VERSION 1 - REVIEW

**Reviewer**	**1**
**Name**	**Brisbois, Ben W.**
**Affiliation**	**University of Northern British Columbia**
**Date**	**23-Jun-2024**
**COI**	**None.**

This article presents a study of adolescent health as viewed by key informants and caregivers in two rural communities with large-scale mines in rural Mozambique. The article is a reasonably well written and appropriately researched qualitative study on a topic of importance to public health. With these strengths in mind, and based on some theoretical and methodological issues, I am recommending the paper be accepted if major revisions are made.

One major factor necessitating a major rewrite of the paper is its attempt to draw conclusions about the factors affecting adolescent health without actually presenting the perspectives of the adolescents themselves. While the authors note this as a limitation and allude to a separate article presenting the results of research with adolescents from the communities in question, it appears methodologically and politically questionable to separate publication of the results in this way. It may be an instance of trying to generate too many publications out of a single data set, although I can see ways to guard against such risks with the help of a greater theoretical engagement, discussed below. I note that the results of the other study appear not to be referred to in the present article, even in the discussion section. Qualitative studies of perceptions of health can very productively triangulate across and among different voices discussing the same health issues, but this

source of rich insights in countless other studies has not been attempted by the authors. For example, the differences between how adolescents view the determinants of their health and how caregivers, service providers, health professionals and other key informants view them are likely extremely illustrative and meaningful in both a social scientific and a health equity sense.

I therefore strongly recommend analyzing how those different voices concur and differ with respect to the health implications of mining in the area. While a single publication presenting the different voices and triangulating across them would be the most efficient way to do this, there are other ways. The present article could refer to the separate publication regarding the adolescents' own perspectives in both the introduction (when framing the study) and in the discussion. Since the study is one of perceptions of health and the first author is a qualitative researcher, it would be helpful and even expected to contextualize the results in relation to the large and rich literature in public health, medical anthropology and possibly risk perception on how understandings of health differ across axes of social difference such as age, gender, racialized identity and – especially – class.

Along similar lines, I suggest that the paper deepen its literature review in a few key areas. The ways in which different types of people understand health and well-being is one of these, as discussed above. It would be helpful to more emphatically frame the study as being less about adolescent health in mining-affected communities than about how the health of adolescents is understood by various categories of people other than the adolescents themselves. There is a large and perceptive literature on how the poor health of youth, poor people, residents of rural areas and racialized groups (among others) is often characterized by relatively privileged people as resulting from the poor choices or inherent characteristics of those marginalized groups. This tends to lead to suggestions for improving health based on educational/behavioural and sometimes technological interventions, which are typically less effective and equitable than more structural interventions targeting upstream social and environmental determinants of health. The present study's focus on sexual health among youth as discussed by caregivers and relatively privileged adults, leading to recommendations for educational interventions, is one of a few elements suggesting that such dynamics are likely at play. A better engagement with relevant literature would help to draw out such insights.

In particular, the literature review in the intro and discussion could also do a better job at contextualizing mining and health in Mozambique with respect to highly relevant structural factors that drive both resource extraction-intensive economic development models and the lack of availability of health services (I note a reference on page 4, lines 66-7 to 'power and structural inequalities' but this is not explained or elaborated on). While I am not an expert in Mozambique or sub-Saharan Africa, I know that there is literature on Mozambique's colonial and postcolonial history that shows how foreign military interventions and macroeconomic reforms compromised its promising approach to primary health care following independence from Portugal and led to decades of austerity measures with

catastrophic implications for access to health services and overall population health (I am thinking of James Pfeiffer's work but there is undoubtedly more available). This insight appears relevant to the authors' recommendations, for example calling on mining companies to provide more resources for health in a voluntary corporate social responsibility kind of way (as opposed to the state providing such resources through adequate taxation and local control over its own model of development and health system operations).

Finally, better use could be made of relevant Mozambican scholarship in relation to the above suggestions. Publishing on health in Mozambique without citing local scholars – including ones publishing in Portuguese or other local languages – is the kind of practice that has been repeatedly criticized in the literature on decolonizing global health.

Some other assorted issues:

• Define 'stakeholder' (in title and keywords) and what the specific nature of the 'stake' they 'hold' is in relation to the adolescents

• You should discuss the possible implications of selection bias in the interview and focus group samples, for example in picking 'community members willing and able to express themselves in a group'. In light of possible sensitivities related to speaking out against an important local employer, what does this suggest in terms of perspectives that may be underrepresented in or excluded from your sample?

• Excluding the interviewee who refused to be recorded 'in order to maintain a high level of consistency of the methodology and analysis' (p 9 lines 213-4) is not actually great qualitative research. Contrasting or extreme cases can be highly illustrative with respect to the themes that emerge in analyzing qualitative data. In this case, the sensitive nature of the topic suggests that the person who didn't want to be recorded might have particularly interesting or 'juicy' things to say about mining's impacts on adolescent health. If you have data for this individual then you should include it in the analysis. If not, this appears to be a questionable methodological decision that should be acknowledged in the limitations.

• Discuss the rationale for having separate men's and women's focus groups, and what this might imply for how themes emerged (the composition of facilitation of focus groups has a strong influence on how data emerges in them).

• The gender breakdown of the key informants appears relevant. For example, in relation to adolescent girls' sexual health, it would be important to note whether this is originating in a largely male set of interviewees (and what the implications of this might be in light of local gender roles). Any differences in themes that emerged across gender of key informants, or between men's and women's focus groups, should be analyzed and discussed in the paper. Other social differences within the group of key informants (class, racialized identity, etc.) would also be important to at least consider as possible factors influencing the themes that emerged. Any differences between the two districts that were studied should also be noted.

• P14 line341: “there were no these types of diseases” appears to have a typo – should this be ‘not’ instead of ‘no’? In the same quote, line 346, what is meant by ‘flour’?

• P15 lines 371-373 ends a section with the following sentence: “Participants from Moma district specified in which circumstances adolescents are exposed, which represents a constant threat to their health and well-being.” What are these circumstances, and how do they threaten health and well-being?

• P16 line 426: what are ‘school corners’?

• There is a quote on page 19 which appears to have a couple of issues to address: “I’m saying that I tried to send a letter, I deliver the letter saying that we have our children that are who might be able to work in the company. We heard they took other people and adolescent end up like this. Now they don’t want us inside [the mining concession area], but these companies are located in our territory.” Is there an ‘s’ missing at the end of ‘adolescent’? What does it mean that adolescents ‘end up like this’? What is the significance of the fact that ‘they don’t want us inside’ the mining concession area. Are people being forcibly kept out of the concession area? This would appear to be relevant for health, well-being and power dynamics in the region. This should be explored / explained.

• P19line 493, ‘planed’ should be ‘planned’

• P21line568, ‘increase’ should be ‘increased’

• The sentence at the end of page 21 and beginning of page 22 is not a complete sentence.

• P22line579 ‘included’ should probably be ‘concluded’?

**Reviewer**	**2**
**Name**	**Chimoyi, Lucy**
**Affiliation**	**The Aurum Institute, Implementation Research**
**Date**	**26-Aug-2024**
**COI**	**None**

This is a very topical problem with wide research gaps. Extractive industries in SSA have huge economic impacts to a community but mostly, there are underserved populations that may be neglected. This research is trying to address the health impacts extractive industries have on adolescents.

I have reviewed this manuscript and have raised the following comments for your consideration.

General comments

- One of the major limitations in this manuscript was that the target group was not considered as a potential stakeholder to be invited to participate in this research.

- Can the authors clarify which age group they classified adolescents in their study as because according to the WHO definition, an adolescent ranged between 10 and 19 years. I'm sure that there could have been caregivers between 18 and 19 years and would be eligible for participation who possible due to unfortunate circumstances were left head households and take care of their younger siblings/relatives.

- What age range did the authors consider as adolescents in the manuscript/study?

- Can the authors share the interview guides?

Introduction

- In South Africa, through their corporate social responsibilities, mining companies have partners with other organizations to reduce the impact of their industry on the communities. What are the mining companies in Mozambique doing to mitigate some of these health concerns affecting the general communities? Do these initiatives benefit the adolescents? Why do you think adolescents are left out and the focus is on adults and children? This is important to note in the introduction as it segues well into the reasons why this study is relevant.

Methods

- The map in figure 1 is very informative and a good ideal. A few suggestions to make the map more informative would be:

o Since there are no provincial names, it may be better to revise tin the legend the name "provinces" to "provincial boundaries"

o Consider a different fill for Moatize and Moma (from a solid fill to a hatch fill)

o Include the following map elements i.e. north arrow and scale to the map.

- Figure two. Use the correct terminology since qualitative is not a study design but a data collection procedure/technique

- Under the study settings, can the authors include the population size of the adolescents according to the latest National Census?

- Data analysis and management: Line 165 – was the I part of the authorship, if yes, include his/her initials as guided by the COREQ guidelines.

- It is not clear whether coding was abductive (confirm whether coding was deductive or inductive) and which framework was applied

Results/Discussion/conclusions

- Line 219 unclear. Is it two groups consisting of one male and one female or three groups (one female and two males)? Revise accordingly.

- Figure 3 in the results is an interesting presentation of the outcome results. My suggestion is that the authors should mention explicitly in the data analysis section of the methods

section, that the “proportions are graphically presented using a radar/spider web chart.” This detail is missing in the methods section.

- Are there no malaria-prevention or general malaria programmes that the mines are supporting in the northern and central regions in Mozambique or are the open pits neglected by the mining companies and therefore increasing the breeding of malaria-causing mosquitoes? In other mines, are the mining companies engaged or supporting any malaria prevention or treatment programmes in these areas through private-public partnership programmes?

- Line 267-271: Are young adolescent girls married off to mineworkers or who are they married off to? Here, I am unable to draw a link between the mining communities and early marriages. Is this not something that is practised in other communities and not very specific to mining communities?

- Line 217-273: Are these inadequate living conditions in their homes?

- Lines 351-352, authors mention that they were unaware of any current mitigation plans to minimize the impact the mines have on health. There was one FGD /KII participant representing the mining community. What were the perceptions of this individual? Are they aware of mitigation plans?

- In the transcripts, did any caregivers report drowning as a public health concern for adolescents? This may be particularly true as the pits created fill up with water and pose significant danger through drowning.

VERSION 1 - AUTHOR RESPONSE

Reviewer #1:

General comments:

This article presents a study of adolescent health as viewed by key informants and caregivers in two rural communities with large-scale mines in rural Mozambique. The article is a reasonably well written and appropriately researched qualitative study on a topic of importance to public health. With these strengths in mind, and based on some theoretical and methodological issues, I am recommending the paper be accepted if major revisions are made.

R1: We thank Reviewer #1 for providing us a positive appraisal with such detailed comments on our manuscript. We have carefully considered the valuable suggestions received to improve the quality of the manuscript further. In the following section, we provide a detailed point-by-point response addressing the comments and suggestions.

One major factor necessitating a major rewrite of the paper is its attempt to draw conclusions about the factors affecting adolescent health without actually presenting the perspectives of the adolescents themselves. While the authors note this as a limitation and allude to a separate article presenting the results of research with adolescents from the communities in question, it appears methodologically and politically questionable to separate publication of the results in this way. It may be an instance of trying to generate too many publications out of a single data set, although I can see ways to guard against such risks with the help of a greater theoretical engagement, discussed below. I note that the results of the other study appear not to be referred to in the present article, even in the discussion section. Qualitative studies of perceptions of health can very productively triangulate across and among different voices discussing the same health issues, but this source of rich insights in countless other studies has not been attempted by the authors. For example, the differences between how adolescents view the determinants of their health and how caregivers, service providers, health professionals and other key informants view them are likely extremely illustrative and meaningful in both a social scientific and a health equity sense.

R2: Thank you for this interesting point and for challenging us on a perspective that we should consider. We agree with your argument regarding the importance of including adolescents' perspectives in our discussion. As you rightly pointed out, it is crucial to include the voices of adolescents when discussing factors affecting their health and well-being. While we acknowledge the limitations of our current study, we would like to clarify that the decision to publish the results separately stems from the aim of focus on the perspectives of caregivers and key stakeholders, which also provides an important context for understanding the broader health issues. We believe that the adolescent-focused data warrants a more detailed and focused discussion due to its unique insights, which is why two (2) separate manuscripts for dedicated analysis were developed. Additionally, the study team put a great deal of efforts to develop adolescent centered and participatory approaches, such as photovoice and role-play, that were exclusively applied to this group. This in our opinion justifies a stand-alone manuscript solely focused on the methodology (https://doi.org/10.1080/16549716.2024.2305506) and results (under revision) of this effort;

To address Reviewer #1 concern, the revised version now includes additional paragraphs in the introductio and discussion chapters that specifically focuses on the perspectives of adolescents themselves, including a references to the published studies (**see lines 126-142; 714-735**). With these additions, we hope to now sufficiently acknowledge the importance of integrating the perspective of adolescents along with ensuring that the connection between the studies is clear. Of note, this additional layer of analysis will be included in the next stage of our ongoing research efforts.

I therefore strongly recommend analyzing how those different voices concur and differ with respect to the health implications of mining in the area. While a single publication presenting the different voices and triangulating across them would be the most efficient way to do this, there are other ways. The present article could refer to the separate publication regarding the adolescents' own perspectives in both the introduction (when framing the study) and in the discussion. Since the study is one of perceptions of health and the first author is a qualitative researcher, it would be helpful and even expected to contextualize the results in relation to the large and rich literature in public health, medical anthropology and possibly risk perception on how understandings of health differ across axes of social difference such as age, gender, racialized identity and – especially – class.

R3: We like this idea very much! We think Reviewer #1 raises an important point, and we strongly agree that the different voices should be considered. To address your recommendation, we have now made revisions to the manuscript **- See response 2**. Since we have written two articles providing methodological and in-depth insights into adolescents, we have revised the manuscript to incorporate these findings more explicitly in this version. The separate publication regarding adolescents' perspectives is referenced in both the introduction (to better frame the study) and the discussion sections, making it clear how the adolescent data complements and expands upon the current findings. (**see lines 126-142; 714-23**).

Along similar lines, I suggest that the paper deepen its literature review in a few key areas. The ways in which different types of people understand health and well-being is one of these, as discussed above. It would be helpful to more emphatically frame the study as being less about adolescent health in mining-affected communities than about how the health of adolescents is understood by various categories of people other than the adolescents themselves. There is a large and perceptive literature on how the poor health of youth, poor people, residents of rural areas and racialized groups (among others) is often characterized by relatively privileged people as resulting from the poor choices or inherent characteristics of those marginalized groups. This tends to lead to suggestions for improving health based on educational/behavioural and sometimes technological interventions, which are typically less effective and equitable than more structural interventions targeting upstream social and environmental determinants of health. The present study's focus on sexual health among youth as discussed by caregivers and relatively privileged adults, leading to recommendations for educational interventions, is one of a few elements suggesting that such dynamics are likely at play. A better engagement with relevant literature would help to draw out such insights.

R4:). We appreciate Reviewer #1's important point and fully agree that more information on mining projects and their impacts, particularly on vulnerable groups like adolescents, is needed. We acknowledge that adolescents perspectives is essential for a comprehensive understanding of their well-being and have enphasized this in the revised version of the manuscript (see response #2).In response to this suggestion, we have revised our Introduction and Discussion sections to reflect these considerations. (**see lines 126-149:714-723**)

In particular, the literature review in the intro and discussion could also do a better job at contextualizing mining and health in Mozambique with respect to highly relevant structural factors that drive both resource extraction-intensive economic development models and the lack of availability of health services (I note a reference on page 4, lines 66-7 to 'power and structural inequalities' but this is not explained or elaborated on). While I am not an expert in Mozambique or sub-Saharan Africa, I know that there is literature on Mozambique's colonial and postcolonial history that shows how foreign military interventions and macroeconomic reforms compromised its promising approach to primary health care following independence from Portugal and led to decades of austerity measures with catastrophic implications for access to health services and overall population health (I am thinking of James Pfeiffer's work but there is undoubtedly more available). This insight appears relevant to the authors' recommendations, for example calling on mining companies to provide more resources for health in a voluntary corporate social responsibility kind of way (as opposed to the state providing such resources through adequate taxation and local control over its own model of development and health system operations).
R5: Thanks for this important observation and providing a relevant reference in the field. In response to your suggestion, we have added a relevant reference to the literature on Mozambique's colonial and postcolonial history, including the work of James Pfeiffer and others (**see lines 88-102**).

Finally, better use could be made of relevant Mozambican scholarship in relation to the above suggestions. Publishing on health in Mozambique without citing local scholars – including ones publishing in Portuguese or other local languages – is the kind of practice that has been repeatedly criticized in the literature on decolonizing global health.
R6: We appreciate the reviewer's emphasis on this issue, especially in the context of decolonizing global health. While efforts have been made recently to address this gap, much of the published literature, including works in local languages, is not always readily accessible. In response to your feedback, we have made an effort to incorporate additional papers from relevant Mozambican scholars in our references, including works published in Portuguese in both Introduction and discussion chapters.

Specific comments:

Define ‘stakeholder’ (in title and keywords) and what the specific nature of the ‘stake’ they ‘hold’ is in relation to the adolescents

R7: Many thanks for pointing this out. We fully share your caution in using the term “Stakeholder” in this study. *“In our research, stakeholders refer to government and private sector representatives, civil society organizations, mining company representatives,caregivers (of adolescents aged 10-19 years) and other local individuals who have an interest in or impact (directly or indirectly) adolescent health and well-being in communities affected by extractive industries. The specific nature of the stake they hold varies according to each group's distinct interests, obligations, and contribution, shaping their engagement in different ways”*. Hence, we clarified and included the above statement this point in the revised manuscript (**see lines 191-196):**

- **Government representatives** are responsible for policies and programs that impact adolescent health and well-being.
- **Private sector** and mining companies influence local economies, environmental conditions, and social structures, which in turn affect adolescent health.
- **Civil society** organizations advocate for adolescent rights, provide services, and monitor industry practices.
- **Local individuals** and communities experience both the direct and indirect effects of extractive activities on their daily lives, education, and health services.

You should discuss the possible implications of selection bias in the interview and focus group samples, for example in picking ‘community members willing and able to express themselves in a group’. In light of possible sensitivities related to speaking out against an important local employer, what does this suggest in terms of perspectives that may be underrepresented in or excluded from your sample?

R8: Thank you for higlighting this ponit, which has helped us critically reflect on the limitations of our study. This limitation has been incorporated into the revised manuscript. It now reads: *“We acknowledge the potential selection bias in our sample, particularly regarding underrepresented perspectives due to sensitivities around a local employer (mining company). Thus, we recognize that some viewpoints may still be underrepresented and suggest future research using ethnographic methods or longitudinal studies to address this”.* (**see lines 802-806**)

Excluding the interviewee who refused to be recorded 'in order to maintain a high level of consistency of the methodology and analysis' (p 9 lines 213-4) is not actually great qualitative research. Contrasting or extreme cases can be highly illustrative with respect to the themes that emerge in analyzing qualitative data. In this case, the sensitive nature of the topic suggests that the person who didn't want to be recorded might have particularly interesting or 'juicy' things to say about mining's impacts on adolescent health. If you have data for this individual then you should include it in the analysis. If not, this appears to be a questionable methodological decision that should be acknowledged in the limitations.

R9: We think Reviewer #1 raises an important methodological point and we strongly agree that including the interview would be much better**.** Indeed**,** we recognize that this decision may have limited our ability to capture certain perspectives, from a stand point of someone whose concerns about protecting information and interests of the company were higtened. Indeed, in this specific case, we initially considered inclusion of this interview; however, there was an additional element additing weight to our final decision. During the interview, the superior of the interviewee unexpectedly joined the conversation creating additional discomfort and hesitation on the side of the respondent. Given this interference, we believe that the responses were likely influenced by external pressure, which compromised the authenticity and reliability of the data.

At the same time, we believe that this approach enhances the depth and rigor of our work and adheres to the principle of data integrity in our analysis. To acknowledge this, we will explicitly mention it as a methodological and ethical limitation in the revised manuscript and supported by the respective references (**see lines 268–271**).

P14 line341: "there were no these types of diseases" appears to have a typo – should this be 'not' instead of 'no'? In the same quote, line 346, what is meant by 'flour'?

R10: We are very grateful for these sharp observations. We have rephrased the statement "there were no these types of diseases" and replaced it with "*these types of diseases did not exist*"**.** Additionally, regarding the term "flour", we have clarified its meaning in the revised text. The sentence now reads: "*Even the white flour [meal prepared with maize (locally called ''xima')] and coal dust are mixed into food [visibly affected by the dust*]." **(*see lines 430 - 434*)**

P15 lines 371-373 ends a section with the following sentence: "Participants from Moma district specified in which circumstances adolescents are exposed, which represents a constant threat to their health and well-being." What are these circumstances, and how do they threaten health and well-being?

R11: We have now revised the paragraph to provide greater clarity on the circumstances discussed by participants, incorporating a specific example. The revised text now reads *[See lines 461-464]*:

"Participants described specific situations in which adolescents are exposed, highlighting the constant threat to the health and well-being. One respondent gave an example of the increase in reported cases of diarrhoeal diseases in the recent years, which they also associated with the negligence of mining projects operating within their communities."

P16 line 426: what are 'school corners'?

R12: In Mozambique, "school corners" are designated safe spaces within educational institutions where students can receive counseling and report any concerns. The programme is established in collaboration with local education authorities and serve as supportive environments for students, particularly focusing on the empowerment and well-being of adolescents. In the revised version the following statement was added (**see lines 739 -748**):

"For instance, school corners play a crucial role in promoting a safe and supportive educational environment, addressing students' concerns, and contributing to their overall well-being. The programme is known for its peer support approach, involving adolescent counsellors and young people, who aim to respond to questions simply and "youthfull. While school corners is the most prominent example of a functioning adolescent health programme in the country, resources for the developing similar programmes for vulnerable groups, such as adolescents residing and working in affected communities remain scarce. Therefore, health and educational interventions that are tailored for adolescents represent a valuabe opportunity to improve teacher- parent-child communication and protect them from making unhealthy choices".

There is a quote on page 19 which appears to have a couple of issues to address: "I'm saying that I tried to send a letter, I deliver the letter saying that we have our children that are who might be able to work in the company. We heard they took other people and adolescent end up like this. Now they don't want us inside [the mining concession area], but these companies are located in our territory." Is there an 's' missing at the end of 'adolescent'? What does it mean that adolescents 'end up like this'? What is the significance of the fact that 'they don't want us inside' the mining concession area. Are people being forcibly kept out of the concession area? This would appear to be relevant for health, well-being and power dynamics in the region. This should be explored / explained.

R13: We really appreciate your careful review. We acknowledge the missing "s" in "adolescent". This has been corrected in the revised manuscript.

In regards to the meaning of "*adolescents end up like this*" as term used by the participant– reflects local concerns about the lack of employment opportunities for young people in mining areas. The statement "they don't want us inside" refers to restrictions placed on local communities regarding access to the mining concession area. While people are not necessarily being forcibly removed, the mining company enforces clear boundaries that prevent local residents from entering these spaces, even though the mines are situated on lands historically occupied by the community. We have now revised the text to clarify these aspects and further discuss their broader implications in the revised manuscript (**see lines 570–576**). *"I'm saying that I tried to send a letter, I deliver the letter saying that we have our children that are who might be able to work in the company. We heard they took other people and adolescents end up like this [refers to local concerns about the lack of employment opportunities for young people in mining areas]. Now they don't want us inside [refers to restrictions placed on local communities regarding access to the mining concession area], but these companies are located in our territory". (FGD with male caregivers, Moatize)*

P19line 493, 'planed' should be 'planned'

R14: We apologize for the typo, and we have now corrected "planed" to "planned" in the revised manuscript. (**see line 587**)

P21line568, 'increase' should be 'increased'

R15: We apologize for the typo, and we have now corrected "increase" to "increased" in the revised manuscript. (**see line 665**)

The sentence at the end of page 21 and beginning of page 22 is not a complete sentence.

R16: Thank you for pointing that out. We have already rephrased and revised the sentence in the new version of the manuscript to ensure clarity and completeness. We appreciate your valuable feedback**. (see lines 639 - 644)**

"A significant concern in Mozambique, one of the countries with the highest HIV prevalence in the world, is the alarming proportion of commercial sex workers who are adolescent girls and minors. This issue is particularly pronounced in rural areas, where limited access to education, healthcare, and economic opportunities exacerbates vulnerabilities".

P22line579 'included' should probably be 'concluded'?

R17: Thank you for pointing out this detail. We have corrected "included" to "concluded" in the revised version of the manuscript. (**see line 676**)

Reviewer #2:

General comments:

One of the major limitations in this manuscript was that the target group was not considered as a potential stakeholder to be invited to participate in this research.

R18: Thank you for this interesting perspective and for challenging us. We agree completely with Reviewer #2 and #1. We fully acknowledge the importance of adolescent-centered perspectives and recognize that their voices provide critical insights into their own health experiences and now is more explicitly discussed (**see response #2**) .

Can the authors clarify which age group they classified adolescents in their study as because according to the WHO definition, an adolescent ranged between 10 and 19 years. I'm sure that there could have been caregivers between 18 and 19 years and would be eligible for participation who possible due to unfortunate circumstances were left head households and take care of their younger siblings/relatives.

R19:. We want to clarify here that in this study, we included caregivers of adolescents aged 10 to 19 years, as per to the WHO. We acknowledge that some caregivers, particularly those aged 18 to 19, may themselves fall within this age range, especially in rural contexts where young people assume caregiving responsibilities due to various social or economic circumstances. Of note, the perspectives of adolescents who themselves were caregivers were captured in main study. To adress the reviwer #2 concern, we have now included a reference of the study component focused on adolescents in method chapter (**see lines 191-196**).

What age range did the authors consider as adolescents in the manuscript/study?

R20: We have now revised and clarified this aspect. In our study, we considered adolescents to be individuals aged 10–19 years, following the WHO definition. We have now explicitly included this definition in the introduction (**see line 70-71**).

Can the authors share the interview guides?

R21: Thank you for this important observation. We have now attached the original interview and focus group discussion guides (in Portuguese) as supplementary material.

Specific comments:

Introduction

- In South Africa, through their corporate social responsibilities, mining companies have partners with other organizations to reduce the impact of their industry on the communities. What are the mining companies in Mozambique doing to mitigate some of these health concerns affecting the general communities? Do these initiatives benefit the adolescents? Why do you think adolescents are left out and the focus is on adults and children? This is important to note in the introduction as it segues well into the reasons why this study is relevant.

R22: Thank you for raising this important aspect! We have now revised and made more explicit and contextualized, particularly in highlighting the gap in adolescent-focused interventions and the need to understand how key stakeholders frame their health. We acknowledge the importance of this issue and have now incorporated a discussion of mining companies' CSR initiatives in Mozambique into the introduction (**see lines 103-109**), and now reads.

"In fact, in Mozambique, some mining companies have engaged in corporate social responsibility (CSR) initiatives aimed at addressing community health concerns, including access to healthcare and environmental impact mitigation. However, these efforts often primarily target adults and younger children, with adolescents frequently overlooked in program design and implementation".

Methods

- The map in figure 1 is very informative and a good ideal. A few suggestions to make the map more informative would be:

o Since there are no provincial names, it may be better to revise tin the legend the name "provinces" to "provincial boundaries"

R23: We like this idea very much.Hence, we have revised the legend to refer to "provincial boundaries" rather than "provinces". We are grateful for the comments and suggestions that have significantly contributed to enhancing the overall quality of our manuscript (**see line 170**).

Consider a different fill for Moatize and Moma (from a solid fill to a hatch fill)

R24: Thank you for your suggestion. To improve visual distinction, we have addressed this in the current version of the manuscript as recommended (**see line 170**).

Include the following map elements i.e. north arrow and scale to the map

R25: Again, thank you for your suggestion. We have included the requested map elements (**see line 170**).

Figure two. Use the correct terminology since qualitative is not a study design but a data collection procedure/technique

R26: Thank you for challenging us on this critical aspect concerning the use of terminologies in qualitative studies. We fully acknowledge your point regarding the distinction between qualitative approaches and study design. While qualitative approaches refer to the set of data collection techniques, the term “qualitative study design” is commonly used in the literature to describe research that employs qualitative data collection methods within a specific design framework. In our case, we intended to highlight the broader research approach that guides the study, which is rooted in qualitative inquiry. That being said, we have updated Figure 2 to reflect more precise terminology and replaced the term “qualitative study design” with “data collection techniques” to more accurately reflect the methodology used in our study. Moroever, the narrative on study design was updated to reflect this concern (**see line 180**).

Under the study settings, can the authors include the population size of the adolescents according to the latest National Census?

R27: Again, thank you for the suggestions. We have now included the population size of adolescents according to the latest National Census. This information has been added and now reads “The total population of Mozambique is approximately 8 million, representing 25% of the total population. Specifically, the population of adolescents in Moma is 81,110, and in Moatize, it is 85,886”. (**see line 162-164**).

Data analysis and management: Line 165 – was the I part of the authorship, if yes, include his/her initials as guided by the COREQ guidelines.

R28: Thank you for your comment. We have clarified all aspects of the data analysis and management process, specifically regarding the involvement of the principal investigator (her) and research assistant who participated in the coding. As per your suggestion, we have now included the principal investigator’s initials (OC) in the manuscript in accordance with the COREQ guidelines. (**see lines 242-254**).

It is not clear whether coding was abductive (confirm whether coding was deductive or inductive) and which framework was applied.

R29: Thank you for this important point, which makes clear that we need to clarify how the coding approach was employed. In the revised version, we have clarified that the coding process followed an abductive approach, grounded in the health outcomes framework..

The following statement was added to reflect the coding process “A matrix was designed by OC and revised by CG, where the rows represented the participants ID and the columns the questions addressed (organised into pre-determined and emerging themes), which oriented a process of abductive coding. *Once all the data were coded, they were summarised in a matrix for each theme and inserted into a matrix using Microsoft Excel* (OC and CG). *This process*

allowed for the development of emergent themes from the data, consistent with an inductive coding process. Four main themes emerged from the analysis, which were analysed based on their contextual importance during the KIIs and FGDs" (see lines 245-250).

Results/Discussion/conclusions

Line 219 unclear. Is it two groups consisting of one male and one female or three groups (one female and two males)? Revise accordingly

R30: We are very grateful for the sharp observations made by the Reviewer #2. We have revised it for better clarity, and now reads:

*"The two FGDs were comprised of one group of females (n=18) and one group of males (n=17)." (**see lines 299-300**)*

Figure 3 in the results is an interesting presentation of the outcome results. My suggestion is that the authors should mention explicitly in the data analysis section of the methods section, that the "proportions are graphically presented using a radar/spider web chart." This detail is missing in the methods section.

R31: We fully agree with Reviewer #2 regarding the need to align with the presentation of the results of the figure 3 with the method. In response to this concern, we have included the statement and now reads *"By comparing the frequency of mentioned health outcomes between KIIs and FGDs and study sites, we aimed to identify any discrepancies or similarities in the perceived health outcomes. Therefore, proportions are graphically presented using a radar web chart (Fig. 3)". (**see lines 262-265**)*

Are there no malaria-prevention or general malaria programmes that the mines are supporting in the northern and central regions in Mozambique or are the open pits neglected by the mining companies and therefore increasing the breeding of malaria-causing mosquitoes? In other mines, are the mining companies engaged or supporting any malaria prevention or treatment programmes in these areas through private-public partnership programmes?

R32: While there may be broader malaria prevention initiatives in the region, our findings did not identify direct engagement by mining companies in malaria-specific programs. This highlights a potential gap in corporate social responsibility efforts related to vector control in mining areas, which could be an important area for further investigation. Additionally, while we did not assess the environmental impact of mining operations on mosquito breeding sites, participants perceived the presence of open pits and stagnant water in mining areas to increase malaria transmission. To address reviewer comments, we added the following statement: *"Thus, collaboration between mining sector and national control programmes might effectively reduce the risk of vector-borne diseases in settings with high prevalence in*

*SSA. However, further research would be valuable in exploring whether private-public partnerships could enhance malaria prevention efforts in these regions". (**see 694-697**)*

Line 267-271: Are young adolescent girls married off to mineworkers or who are they married off to? Here, I am unable to draw a link between the mining communities and early marriages. Is this not something that is practised in other communities and not very specific to mining communities?

R33: This is an important point, and we fully acknowledge the need for caution in linking early marriages directly to the presence of mineworkers. Our findings suggest that economic factors in the studied mining areas exhacerbate this practice in two ways. On the one hand, participants reported that young adolescent girls, due to financial pressures, engage in risky behaviors such as early sexual debut with individuals who have relatively stable incomes, including those benefiting directly and indirectly from mining activities. On the other hand, some participants perceived that caregivers encourage early marriages as a means to alleviate financial burdens on households. Of note, the study is higlighting the perception of those who experienced this reality, which literature corroborates with. To address reviewer #2 concern we have now included the folowing statement *"While early marriage is not unique to mining communities in Mozambique, the economic disparities and livelihood opportunities created by the presence of industrial mining are perceived to enhance existing social dynamics, including marriage practices among adolescents."* (**see lines 705-708**)

Line 217-273: Are these inadequate living conditions in their homes?

R34: The inadequate living conditions described refer to both household-level challenges and broader structural conditions within mining communities. These inadequate living conditions, combined with multiple disadvantages and the precarious socio-economic circumstances faced by adolescents, were perceived as key factors influencing their sexual and reproductive health outcomes. We have deleted the term "inadequate living conditions" and revised the paragraph to ensure a more precise and comprehensive description in the results. *And now reads, "The lack of stable support systems and economic opportunities is perceived to further exacerbate their exposure to early sexual debut, transactional relationships, and early marriages, as highlighted by participants in our study". Consequently, inadequate living conditions coupled with the multiple disadvantages and precariousness experienced by adolescents was perceived as an important aspect affecting adolescents' sexual and reproductive health (**see lines 353-358**).*

Lines 351-352, authors mention that they were unaware of any current mitigation plans to minimize the impact the mines have on health. There was one FGD /KII participant

representing the mining community. What were the perceptions of this individual? Are they aware of mitigation plans?

R35: Our study could not capture the participant representing the mining community in Moatize distric as mining companies did not answer our invitation to participate in the study. Overall, the FGDs and other key informants in the districts did not mention any concrete programs or strategies in place to address adolescent health concerns directly associated with mining. However, we could not confront these perspectives, as the mining representatives' insights in Moatize district were not captured in this study. We have now included this information in the revised manuscript. (**see lines 440-441**)

In the transcripts, did any caregivers report drowning as a public health concern for adolescents? This may be particularly true as the pits created fill up with water and pose significant danger through drowning

R36: Thank you for this interesting perspective. In our study, caregivers and KII did not explicitly mention drowning as a health concern for adolescents in the study mining areas. However, we recognize that open pits created by mining activities can accumulate water, posing potential drowning risks, especially for children and adolescents. While participants in our study did not raise this issue, it remains a significant environmental and public health concern. Future studies could further explore drowning and other major health risks affecting adolescents, particularly in relation to disability-adjusted life years (DALYs). In the revised version of the manuscript, this aspect is now emphasised in the conclusion chapter: "*Additionally, targeted interventions focusing on education, economic empowerment, and environmental safety are essential to mitigate the identified risks, as well as other potential hazards, such as drowning and injuries, which require further exploration. Future research should investigate the broader impact of these challenges on adolescents' health to inform the development of effective policies and interventions*." (**see lines 749-753**)

VERSION 2 - REVIEW

**Reviewer**	**2**
**Name**	**Chimoyi, Lucy**
**Affiliation**	**The Aurum Institute, Implementation Research**
**Date**	**08-May-2025**
**COI**	

The Authors have addressed all the questions that were raised adequately.
